# Recent progress in DNA methyltransferase inhibitors as anticancer agents

**DOI:** 10.3389/fphar.2022.1072651

**Published:** 2022-12-16

**Authors:** Zhixiong Zhang, Guan Wang, Yuyan Li, Dongsheng Lei, Jin Xiang, Liang Ouyang, Yanyan Wang, Jinliang Yang

**Affiliations:** ^1^ State Key Laboratory of Biotherapy and Cancer Center, National Clinical Research Center for Geriatrics, Innovation Center of Nursing Research, West China Hospital, and Collaborative Innovation Center of Biotherapy, Sichuan University, Chengdu, China; ^2^ Targeted Tracer Research and Development Laboratory, Institute of Respiratory Health, Frontiers Science Center for Disease-related Molecular Network, West China Hospital, Sichuan University, Chengdu, China; ^3^ School of Physical Science and Technology, Electron Microscopy Center of Lanzhou University, Lanzhou University, Lanzhou, China; ^4^ Science and Technology Department, National Clinical Research Center for Geriatrics, West China Hospital, Sichuan University, Chengdu, China

**Keywords:** epigenetic, DNA methylation, DNA methyltransferase, DNA methyltransferase inhibitor, tumor immunotherapy

## Abstract

DNA methylation mediated by DNA methyltransferase is an important epigenetic process that regulates gene expression in mammals, which plays a key role in silencing certain genes, such as tumor suppressor genes, in cancer, and it has become a promising therapeutic target for cancer treatment. Similar to other epigenetic targets, DNA methyltransferase can also be modulated by chemical agents. Four agents have already been approved to treat hematological cancers. In order to promote the development of a DNA methyltransferase inhibitor as an anti-tumor agent, in the current review, we discuss the relationship between DNA methylation and tumor, the anti-tumor mechanism, the research progress and pharmacological properties of DNA methyltransferase inhibitors, and the future research trend of DNA methyltransferase inhibitors.

## 1 Introduction

DNA methylation is a major covalent modification occurring at the C-5 position of cytosine in CpG dinucleotides, with cofactor S-adenosyl-l-methionine (SAM) as a methyl donor, which suppresses the transcription of the methylated gene (Costa et al., 2017). DNA methylation plays an important role in maintaining normal cell function and embryonic development. Once the modification is abnormal, it leads to the occurrence of tumor. Abnormal DNA methylation in a tumor cell is manifested in the following three aspects: 1) DNA methyltransferase (DNMT) is overexpressed and its catalytic activity is enhanced in the tumor cell. 2) The DNA methylation level near highly repetitive sequences in the tumor cell is significantly reduced, resulting in chromosomal instability and reactivation of transposon elements. 3) The tumor suppressor gene (TSG) cannot be normally expressed due to hyper methylation in the promoter (Pechalrieu et al., 2017).

DNA methylation is mediated by DNMT in mammals. Currently, five types of DNMT have been reported, among which DNMT1 and DNMT3A/3B are related to DNA methylation (Masaki et al., 1999; Goffin and Eisenhauer, 2002). Inhibition of DNMT by the DNA methyltransferase inhibitor (DNMTi) is of great significance for tumor immunotherapy. On one hand, the DNMTi can promote tumor antigen presentation by upregulating the expression of tumor-associated antigen (TAA). On the other hand, the DNMTi can enhance the function of cytotoxic T lymphocyte (CTL) by maintaining or upregulating the expression of IL-2, IFN-γ, and other anti-tumor cytokines through demethylation. The DNMTi can also promote immune response by promoting NK cell-mediated cytotoxic effect and modulating immunosuppressive cells. In addition, the DNMTi can remodel the chromatin of tumor cells by inhibiting DNA methylation and histone modification.

Up to now, there are nearly 70 pipelines of DNMTi development worldwide, among which azacitidine, decitabine, clofarabine, and arsenic trioxide have been approved for the treatment of hematological tumors. A few DNMTis have entered the clinical stage for anti-tumor research. However, most of them are still in the pre-clinical stage. In the current review, we discussed the relationship between DNA methylation and tumor, the research progress, mechanism, and pharmacological properties of DNMTis with the purpose of promoting the development of DNMTis as an anti-tumor agent.

## 2 DNA methylation and tumor

### 2.1 Process and mechanism of DNA methylation

**FIGURE 1:**
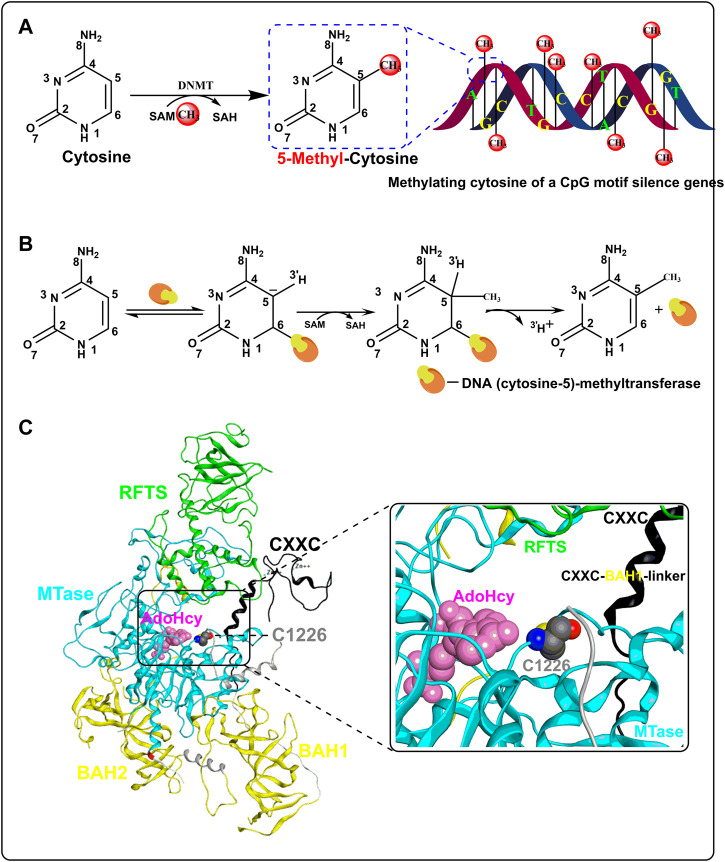
Process and mechanism of DNA methylation. **(A)** The overall process of CpG methylation by DNMT. **(B)** Mechanism of DNA methylation in DNA (cytosine-5)-methyltransferase. **(C)** Crystal structure of human DNA methyltransferase 1 with AdoHcy (PBD code: 4WXX). The RFTS, CXXC, MTase, and BAH domains are colored in green, black, light blue, and yellow, respectively. SAH is colored in pink and residue C1226 is colored in gray.

In a broad sense, DNA methylation refers to the chemical modification of methyl binding to a specific base on DNA through a covalent bond, which may occur at the C-5 position of cytosine, N-6 position of adenine, and N-7 position of guanine (Ahmad and Rao, 1996). Strictly speaking, DNA methylation in common research refers to the process in which the methyl group of SAM is transferred to the C-5 position of cytosine in the 5′-CpG-3′ dinucleotides (CpGs) under the function of DNMTs to form 5-methyl cytosine (Figure 1A). DNA methylation is mediated by DNMT and five DNMTs have been found so far. DNMT1 is mainly involved in the maintenance of the methylation state and the *de novo* methylation of the non-CpG site. DNMT3 comprises DNMT3A, DNMT3B, and DNMT3L, in which DNMT3A and 3B are *de novo* DNA methylation enzymes, while DNMT3L is the regulator of DNMT3A/3B. DNMT2 has no catalytic activity and its function in DNA methylation is unclear (Margot et al., 2001).

The mechanism of DNA methylation was first reported in prokaryotic DNA (cytosine-5)-methyltransferase (Wu and Santi, 1987). First, the C atom of cytosine is flipped out of the DNA double helix and embedded in the catalytic domain when DNA (cytosine-5)-methyltransferase binds to DNA. Second, mercaptan of cysteine at the active site of DNA (cytosine-5)-methyltransferase forms a covalent bond with cytosine C6 through nucleophilic action and simultaneously activates C5. Third, SAM is extremely unstable for methyl binding to the S atom. With the action of DNA (cytosine-5)-methyltransferase, methyl is transferred to activated C5 from SAM, which becomes S-adenosyl-Homocysteine (SAH) later. Finally, with the release of protons at C5 and the breaking of the covalent bond at C6, the process of DNA methylation is completed (Figure 1B). In addition, Zhang et al. (2015) resolved the crystal structure of human DNMT1 in complex with SAH (PDB code: 4WXX) in 2015, which helped us understand the mechanism of DNA methylation catalyzed by DNMT in eukaryotes. Human DNMT1 mainly contains the RFTS domain, the CXXC domain, the MTase domain, and two BAH domains (Figure 1C). SAH is mainly bound in the cavity enclosed by the RFTS, CXXC, and MTase domains, which are also the pockets where DNA binds. Structural analysis showed that when human DNMT1 was not bound to DNA, the RFTS domain could bind to the MTase domain directly through a hydrogen bond and bind indirectly through a CXXC–BAH1 linker, thus forming an auto-inhibitory conformation. However, when DNMT1 binds to unmethylated DNA, the activity of human DNMT1 is inhibited by the CXXC–BAH1 linker. The inhibitory activity of the CXXC–BAH1 linker on human DNMT1 mainly showed two aspects. On one hand, the CXXC–BAH1 linker occupies the DNA binding site and pushes the residue C1226, which is important for the activity of human DNMT1, away from the DNA binding pocket through steric hindrance. On the other hand, the CXXC–BAH1 linker undergoes a helix-to-loop conformational transition and is positioned along the catalytic cleft of DNMT1 to inhibit the interaction between DNMT1 and DNA. Upon binding to the hemi-methylated DNA and SAM, the auto-inhibitory state of DNMT1 was released with the conformation change of the RFTS domain and CXXC–BAH1 linker Zhang et al.,2015). At that moment, the thiol of C1226 in the MTase domain flips over to cytosine and initiates a nucleophilic addition reaction to the C-6 atom of cytosine to form a covalent bond (Figure 1C). The unstable methyl group on SAM was transferred to the C-5 atom of cytosine and then the proton and DNMT are shed from cytosine to form 5-methylcytosine.

### 2.2 The role of DNA methylation in the development of cancer

Hypomethylation plays an important role in the initiation, progression, invasion, and metastasis of different types of cancer. Normal cells ensure genomic stability by silencing satellite sequences through DNA hypermethylation, which is reversed in tumor cells. The loss of DNA methylation of satellite-2 and satellite-α is associated with early breast cancer development, while in ovarian cancer, they promote tumor progression (Widschwendter et al., 2004; Costa et al., 2006). The melanoma-associated cancer-testis (CT) antigen (MAGE) is reactivated due to DNA hypomethylation in melanoma and colorectal cancer, which promotes the development of melanoma and colorectal cancer (Kim et al., 2006). Loss of DNA methylation in the CDH3 gene promoter results in the overexpression of P-cadherin in colorectal and breast carcinomas, which decreased cell polarity and promotes the invasion and migration of colorectal and breast cancer cells (Paredes et al., 2005; Milicic et al., 2008). Overexpression of P-glycoproteins involved in drug resistance in breast cancer patients due to DNA hypomethylation is associated with the advanced tumor stage (Chekhun et al., 2006).

Hypermethylation also plays an important role in the initiation, progression, invasion, and metastasis of different types of cancer. For example, cell adhesion genes CDH1 in gastric cancer cells and CDH13 in primary non-small cell lung cancer cells are silenced due to hypermethylation, which promotes the invasion and metastasis of both types of tumor cells (Seuk Kim et al., 2005; Huang et al., 2015). Various genes associated with DNA repair processes are also hypermethylated in tumor tissues; hypermethylation of the MLH1 gene in gastric cancer leads to abnormal DNA mismatch repair and promotes the progression of gastric cancer (Fleisher et al., 2001). In addition, the apoptosis pathway mediated by death-associated protein kinase 1 (DAPK1)-TMS1 promotes tumor cell apoptosis. The silencing of TMS1 due to hypermethylation promotes the proliferation of breast cancer cells, thus promoting the progression of breast cancer (Gordian et al., 2009).

Taken together, the abnormal methylation of tumor cells is mainly characterized by genome-wide hypomethylation and local hypermethylation. A low level of DNA methylation is associated with the activation of proto-oncogenes. DNA hypomethylation is commonly found in solid tumors and the degree of DNA methylation increases with the increase in the malignant degree. A high level of DNA methylation is associated with the silence of TSG (Akhavan-Niaki and Samadani, 2013). In addition, the ways of DNA hypermethylation in tumor cells are varied. Hypermethylation of multiple genes can occur simultaneously in a tumor, such as APC, DKPK1, MGMT, P16, and RASSF1 in lung cancer, while others, like RASSF1 and P16, could be hypermethylated in a variety of tumors (Castro et al., 2010).

### 2.3 Antitumor mechanism of DNA methyltransferase inhibitors

**FIGURE 2:**
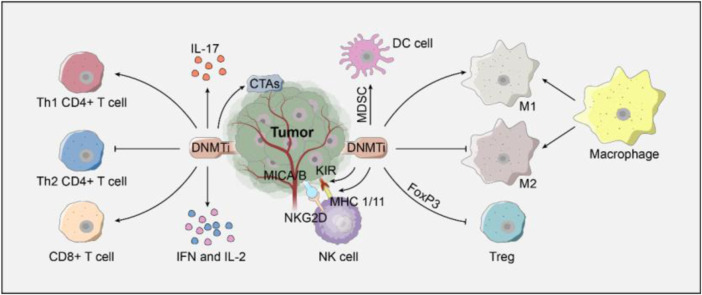
Regulation of DNMTis on immune cells.

DNMTis can enhance the immunogenicity of tumor cells (Figure 2). For example, DNMTis can upregulate the major histocompatibility complex I (MHC-I) by inhibiting DNA hypermethylation in the promoter of MHC-I, thus improving the presentation of TAA and enhancing immunogenicity (Gallagher et al., 2017). In addition, DNMTis can promote the expression of the cancer testis antigen, which can help host CTL to distinguish tumor cells from healthy cells (Gallagher et al., 2017).

DNMTis can enhance the cytotoxic effect of CD8^+^ T cells and help CD4^+^ T cells and NK cells by inducing the expression of immune-stimulatory cytokines (Figure 2). When CD8^+^ T cells were exposed to TAA, DNMTis enhanced the cytotoxic activity of CTL by inhibiting methylation and promoting IL-2 expression (Qiu et al., 2017). When immature CD8^+^ T cells were transformed into effector T cells, DNMTis also induced the demethylation of the three hypermethylated CpG islands in the upstream of IFN-γ, thus enhancing the bioactivity of CTL (Lucarini et al., 2017). The DNMTi promotes CD4^+^ T cells to differentiate into Th1 CD4^+^ T cells by promoting the secretion of IL-17. The killer cell immunoglobulin-like receptor (KIR) on the surface of NK cells can express normally under the demethylation of the DNMTi, which promotes the recognition of abnormal tumor cells after binding to MHC-I specifically (Küçük et al., 2016).

DNMTis can also promote the immune response by regulating the bioactivity of immunosuppressive cells (Figure 2). On one hand, the DNMTi inhibits the differentiation of T lymphocytes into regulatory T cells by regulating the expression of Foxp3 (Costantini et al., 2013). The DNMTi can also inhibit the immunosuppressive effect of myeloid-derived suppressor cells and induce their differentiation into dendritic cells (Zhou et al., 2017). M1 macrophages display anti-tumor phagocytic properties, whereas M2 macrophages have pro-tumorigenic properties. On the other hand, DNMTis can promote the differentiation of macrophages into M1 rather than M2 macrophages by regulating the inflammatory response.

## 3 DNA methyltransferase inhibitors

Currently, there are nearly 70 DNMTi pipelines under development worldwide. In total, 16 pipelines have already been in the clinical research stage and some of them have already entered the phase III clinical study (Table 1). Azacitidine, decitabine, clofarabine, and arsenic trioxide have been approved for the treatment of myelodysplastic syndrome (MDS) and hematological cancer.

**TABLE 1:** Progress of DNMTis in clinical anti-tumor research.

DNMTi	Indications	Phase	NCT number
Guadecitabine	Metastatic colorectal cancer	Phase I	NCT01966289
	Small-cell lung cancer	Phase I	NCT03085849
	Ovarian cancer	Phase II	NCT01696032
	Germ cell cancer	Phase I	NCT02429466
	Paraganglioma	Phase II	NCT03165721
	Acute myeloid leukemia	Phase III	NCT02920008
	Myelodysplastic syndrome	Phase II	NCT02197676
	Hepatocellular carcinoma	Phase II	NCT01752933
RX-3117	Metastatic pancreatic cancer	Phase I/II	NCT03189914
	Metastatic bladder cancer	Phase I/II	NCT02030067
5-Fluoro-2′-deoxycytidine	Head and neck neoplasms, lung cancer, and breast cancer	Phase II	NCT00978250
	Acute myeloid leukemia	Phase I	NCT01041443
	Myelodysplastic syndrome		
5, 6-Dihydro-5-azacytidine	Pleural malignant mesothelioma	Phase I	NF^a^
	Disseminated malignant melanoma	Phase II	NF^a^
Cladribine	Brain and central nervous cancer	Phase I	NCT00019071
	Acute myeloid leukemia	Phase II	NCT02096055
	Lymphoma	Phase II	NCT00053027
	Myelodysplastic syndrome	Phase I/II	NCT02044796
Fludarabine	Head and neck cancer	Phase I	NCT01310179
	Myeloma	Phase I/II	NCT00943319
	Ovarian cancer, fallopian tube carcinoma, and primary peritoneal	Phase I	NCT02118285
	Breast cancer	Phase II	NCT00429572
	Metastatic melanoma	Phase II	NCT00328861
	Cervical cancer and vaginal cancer	Phase II	NCT00005941
	Non-Hodgkin’s lymphoma	Phase II	NCT00597519
	Plasma cell cancer	Phase II	NCT00619645
	Or pharyngeal cancer	Phase II	NCT01585428
	Renal cell cancer	Phase I/II	NCT00005851
	Small intestine cancer	Phase I/II	NCT00416351
	Lymphoma	Phase I/II	NCT00156013
Fazarabine	AML and CMML	Phase I	NF^a^
	Non-small-cell carcinoma	Phase II	NF^a^
	Pediatric solid cancer	phase I	NF^a^
	Metastatic colon carcinoma	Phase II	NF^a^
	Colorectal and ovarian Cancer	Phase II	NF^a^
	Pancreatic carcinoma	Phase II	NF^a^
	High-grade gliomas	Phase II	NF^a^
	Advanced head and neck cancer	Phase II	NF^a^
Procaine	Nasopharyngeal cancer	Phase II	NCT02735317
EGCG	Prostate cancer	Phase II	NCT00676780
	Colon cancer	Early phase I	NCT02891538
	Lung cancer	Phase II	NCT02577393
	Uterine fibroids	Phase I	NCT04177693
	Leiomyoma	Phase II	NCT01311869
Hydralazine	Breast cancer	Phase I/II	NCT00575978
	Ovarian cancer	Phase III	NCT00533299
	Rectal cancer	Phase I/II	NCT00575640
	Lung cancer	Phase I	NCT00996060
	Cervical cancer	Phase II	NCT00404326
Genistein	Breast cancer	Phase II	NCT00244933
	Prostate cancer	Phase II	NCT00058266
	Non-small cell lung cancer	Phase I/II	NCT01628471
	Colon cancer and rectal cancer	Phase I/II	NCT01985763
	Endometrial cancer	Phase I	NCT00099008
	Pancreatic cancer	Phase II	NCT00882765
	Bladder cancer	Phase II	NCT01489813
	Lymphoma	Phase II	NCT02624388
	Kidney cancer	Early phase I	NCT00276835
Equol	Breast cancer	Early phase I	NCT02352025
Curcumin	Colorectal cancer	Phase II	NCT02439385
	Breast cancer	Phase II	NCT01042938
	Head and neck cancer	Early phase I	NCT01160302
	Pancreatic cancer	Phase II	NCT00192842
	Small lymphocytic lymphoma	Phase II	NCT02100423
Disulfiram	Metastatic breast cancer	Phase II	NCT04265274
	Prostate cancer	Phase I	NCT02963051
	Non-small-cell lung cancer	Phase II/III	NCT00312819
	Germ cell tumor	Phase II	NCT03950830
	Multiple myeloma	Phase I	NCT04521335
	Melanoma	Phase II	NCT02101008
	Glioblastoma	Early phase I	NCT01907165
Resveratrol	Colon cancer	Phase I	NCT00256334
	Liver cancer	Phase I/II	NCT02261844
	Lymphangioleiomyomatosis	Phase III	NCT03253913
Caffeic acid	Esophageal cancer	Phase III	NCT04648917

^a^ Not found (NF) the NCT number in the website https://clinicaltrials.gov.

### 3.1 DNA methyltransferase inhibitors approved for marketing

#### 3.1.1 Cytarabine derivant: 5-Azacitidine

5-Azacitidine (**1**) is a cytarabine derivative synthesized in 1964. It was not clear until 1980 that it exerts anti-tumor activity by inhibiting DNA methylation (Jones and Taylor, 1980). 5-Azacitidine is modified by nucleotide reductase and then phosphorylated by uridine cytidine kinase to form a triphosphate, 5-azacitidine triphosphate, which is integrated into the DNA sequence as a cytosine analog during DNA replication, thereby inhibiting DNA cytosine methylation (Rius et al., 2009). It can also cause the degradation of DNMT1 by binding to the sulfhydryl of DNMT1 with a covalent bond (Ghoshal et al., 2005). 5-Azacitidine was approved by the Food and Drug Administration (FDA) for the treatment of MDS in 2004 and it was the first DNMTi in clinical practice with a trade name of Vidaza (Kaminskas et al., 2005). Studies showed that 5-azacytidine could inhibit the L1210 cell with ID50 and ID90 of 0.019 and 0.15 μg/ml *in vitro*, respectively (Li et al., 1970). In addition, a study showed that 5-azacitidine can significantly increase the tri-methylation of lysine-4 on histone H3 in mouse primary spermatocytes, which indicated that 5-azacitidine could modulate histone methylation (Burlibaşa et al., 2021). It could also reduce tumor growth in two intracerebral glioblastoma models *in vivo*.

**FIGURE 3:**
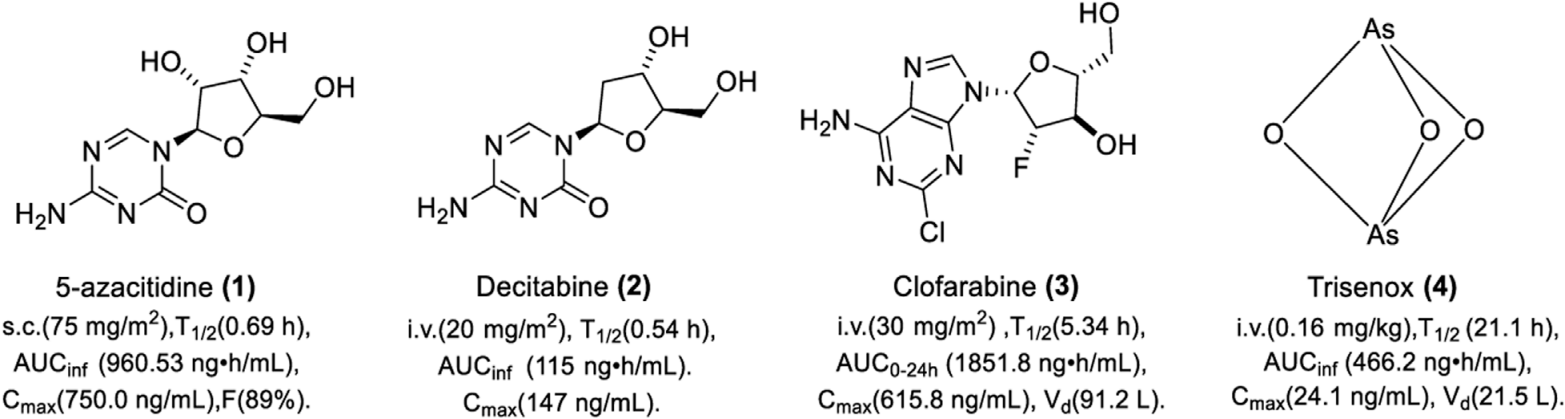
Chemical structure of DNMTis approved by the FDA.

5-Azacytidine is mainly used for the treatment of MDS and hematologic tumors clinically. In the clinical trial “CALGB 8921,” 72 patients with refractory anemia with excessive blasts, chronic myelomonocytic leukemia (CMML) and acute myeloid leukemia (AML), were subcutaneously administered 75 mg/m^2^ of azacitidine every 4 weeks for 7 consecutive days. The overall response (OR) was 13.9%. The mean and median durations of partial response were 810 and 430 days, respectively; 80% of patients were still in partial response (PR). Severe side effects, mainly including thrombocytopenia, febrile neutropenia, and fever, were observed. As shown in Figure 3, the pharmacokinetics (PK) of azacitidine was studied in six MDS patients. A single dose of 75 mg/m^2^ of azacitidine was intramuscularly administered on day 1 (Marcucci et al., 2013). The absolute bioavailability of azacitidine for subcutaneous administration is 89%. The dose of 75 mg/m^2^ is well-tolerated and it could be rapidly absorbed and widely distributed. It is easy for hydrolysis and deamination in plasma, which results in a short half-life. The median half-lives of azacitidine were 0.36 ± 0.02 h and 0.69 ± 0.14 h for subcutaneous and intravenous administration, respectively. Urinary excretion is the primary route of elimination of 5-azacitidine and its metabolites. Vidaza is currently administered subcutaneously for high-risk MDS, CMML, and AML. The recommended dose for the first treatment cycle is 75 mg/m^2^, administered subcutaneously daily for 7 consecutive days.

#### 3.1.2 Deoxycytidine derivant: Decitabine

Decitabine (**2**) was approved by the FDA in 2006 for the treatment of MDS with a trade name of Dacogen (Center for Drug Evaluation and Research). Because it is deoxy, decitabine can be converted directly to a cytosine analog (5-azabine triphosphate) by deoxy-cytidine kinase without prior deoxidation. The inhibitory activity of DNMT is 30 times more than that of azacitidine (Rius et al., 2009). High concentrations of decitabine can induce cell death by forming non-functional DNA during DNA replication, while low concentrations of decitabine can bind DNMT covalently, which leads to DNMT inactivation without causing cell death (Momparler, 2005). Studies showed that decitabine could inhibit the proliferation of SNU719, NCC24, and KATOIII cells *in vitro* (Nakamura et al., 2016). In addition, a study showed that decitabine could enhance apoptosis induced by histone deacetyltransferase inhibitor (HDACi) depsipeptide in human thoracic cancer cells. Further study showed that decitabine increased the depsipeptide-induced acetylation of histone H3 and H4 (Zhu and Otterson, 2003). Decitabine administered once daily at 1 mg/kg for 5 days could result in the regression of EL4 tumor growth in C57BL/6 mice *in vivo* (Wang et al., 2013). Decitabine is prone to deamination and causes granulocytopenia, which limits its clinical application.

Decitabine was mainly used for the treatment of MDS and cancer in clinical practice. Patients with MDS intravenously receive 20 mg/m^2^ of decitabine for 1 h daily and continuously receive the same dose for 5 days; the complete response (CR) and OR was 20% and 61%, respectively. The overall survival (OS) was 20 months. The main side effects are neutropenia, nausea, and fatigue. The PK was evaluated in 11 patients, who intravenously received 20 mg/m^2^ for over 1 h, and plasma concentration–time profiles after discontinuation of infusion showed a biexponential decline. As shown in Figure 3, decitabine can be absorbed rapidly. Upon repeat doses, there was no systemic accumulation or any changes in PK parameters. The deamination of cytidine deaminase in liver, intestinal epithelial cells, granulocytes, and whole blood is the main metabolic pathway of decitabine. The recommended dose of decitabine for its indication of MDS is 15 mg/m^2^ with continuous intravenous infusion for at least 3 h, once every 8 h for 3 consecutive days.

#### 3.1.3 Purine nucleoside derivant: Clofarabine

Clofarabine (**3**) is a purine nucleoside DNMTi, which was approved by the FDA in 2004 for the treatment of refractory or relapsed AML in children with the trade name of clofarabine. A study showed that clofarabine inhibited the proliferation of tumor cells by downregulating DNMT1 and inhibiting the methylation of TSG, such as PTEN, APC, and RARβ2 (Majda et al., 2010). It can also promote the expression of p21, which competitively binds to the binding site of DNMT1 on the proliferating cell nuclear antigen (PCNA) protein, thus blocking the activity of the PCNA-promoting DNMT1 on hemimethylated DNA (Iida et al., 2010). In another study, clofarabine and fludarabine showed a synergistic effect against AML cells by increasing the methylation of lysine 4/9/27/36/79 on histone H3 (Valdez et al., 2011).

Clofarabine (20, 30, and 40 mg/m^2^/day) was administered intravenously for 5 days in patients with relapsed AML or elderly patients with newly diagnosed AML (Tatsuya et al., 2013). The OR was 43% with four (29%) patients achieving complete remission, and two (14%) patients achieved incomplete remission. The maximum tolerated dose (MTD) was 30 mg/m^2^/day and the most common adverse events were thrombocytopenia and anemia. As shown in Figure 3, clofarabine (30 mg/m^2^/day by i.v. infusion) was absorbed rapidly with a narrow distribution. The C_max_ and exposure in 24 h increased with increasing dose. Kidney excretion is the main way for clofarabine elimination. For patients with relapsed or resistant AML who received at least two previous treatments, the recommended therapy for clofarabine is 52 mg/m^2^/d over 2 h for 5 consecutive days, repeated every 2–6 weeks after organ function has recovered to baseline levels.

#### 3.1.4 Arsenical agent: Arsenic trioxide

Arsenic trioxide (**4**) was approved by the European Commission in 2002 for the treatment of relapsed or persistent acute promyelocytic leukemia (APL) with the trade name of Trisenox (Cohen et al., 2001). Studies showed that Trisenox could promote the apoptosis of leukemia cells by inhibiting DNA methylation (Badarkhe et al., 2016). However, the mechanism of DNA methylation inhibition is unclear. Interestingly, studies showed that arsenic trioxide could reduce global acetylation of lysine 16 on histone H4 by binding to histone acetyltransferase hMOF in HeLa cells and HEK293T cells, while also increasing deacetyltransferase HDAC4 expression (Liu et al., 2015). These data provide a new theoretical basis for elucidating the mechanism of As toxicity.

Five patients with newly diagnosed APL were intravenously administered at a dose of 0.16 mg/kg for 40 min infusion on the first day followed by 18–20 h daily at a very slow rate with an infusion speed of 8 drips/min (Gao et al., 2018). All patients achieved hematologic complete remission after induction therapy, but also experienced side effects such as abnormal coagulation and anemia. As shown in Figure 3, Trisenox was absorbed slowly with a low clearance rate and a long half-life, which results in wide distribution in tissues. When the drug was stopped, the arsenic content in tissues was detected from high to low in the order of skin, ovary, liver, and kidney. During the treatment, the amount of arsenic excreted in urine in 24 h was 1%–8% of the dosing dose. Concentrations of total arsenic were constant throughout the administration period at a steady state. The PK remained the same during continuous administration. The clearance rate of continuous slow infusion is slower than that of rapid infusion, which facilitates to maintain the effective concentration at a low level and avoid the toxicity caused by a high concentration. It seems that hepatic toxicity is the main reason leading to the termination of Trisenox. For patients with newly diagnosed or relapsed APL, the recommended dose is 0.15 mg/kg intravenously daily in combination with tretinoin until bone marrow remission and not to exceed 60 days in the induction cycle.

### 3.2 DNA methyltransferase inhibitors in clinical research

#### 3.2.1 Dinucleotide derivant: Guadecitabine

Guadecitabine (SGI-110, **5**) is a dinucleotide derivative of decitabine, which inhibits DNA methylation by the same mechanism of decitabine, but its PK and stability are superior to those of decitabine due to the phosphodiester bond of SGI-110, which can resist the transient degradation of cytidine deaminase. Studies showed that SGI-110 reduced the methylation of T24 and HCT116 cells and caused the depletion of DNMT1 *in vitro* (Yoo et al., 2007). The study also showed that treatment with SGI-110 or in combination with HDAC inhibitor MS275 could reverse the epithelial mesenchymal transition (EMT) by inducing tri-methylation of lysine-27 on histone H3 (H3K27me3) in triple-negative breast cancer (TNBC) cells. The level of H3K27me3 in breast cancer patients was positively correlated with their survival. These data suggested that SGI-110 alone or in combination with the HDAC inhibitor could improve the survival of breast cancer patients (Su et al., 2018). In addition, SGI-110 could reduce DNA methylation in the promoter of the p16 gene in a human xenotransplantation tumor model *in vivo*.

**FIGURE 4:**
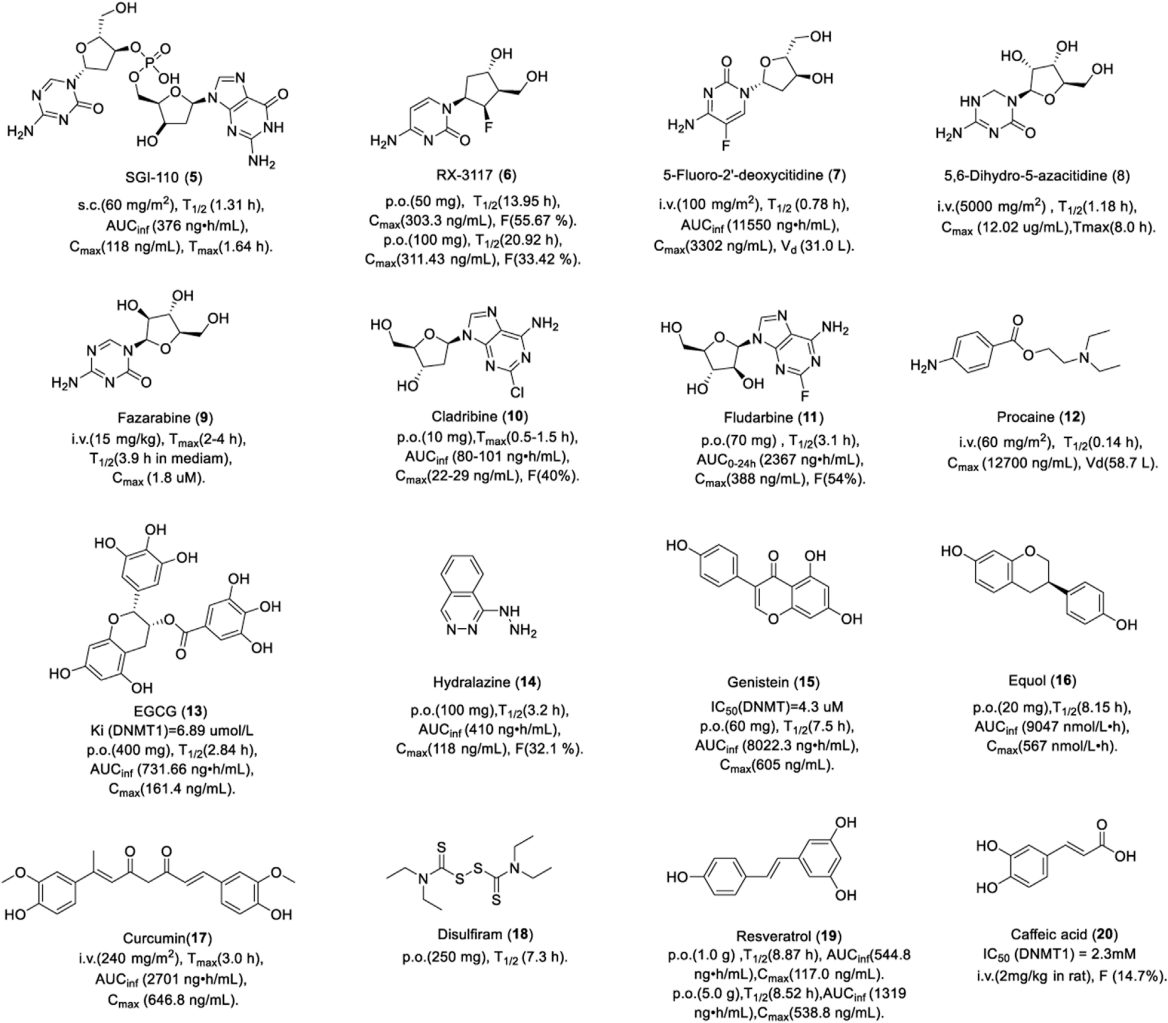
Chemical structure of DNMTis in clinical.

SGI-110 is used for the treatment of MDS and AML in clinics. In total, 74 patients with AML and 20 patients with MDS were randomly assigned to subcutaneous SGI-110 with a dose of 3–125 mg/m^2^, either once daily for 5 consecutive days (daily×5), or once weekly for 3 weeks. The median OS for patients with AML and MDS was 140 days and 282 days, respectively (Issa et al., 2015). No dose-limiting toxicity (DLT) was noted up to 90 mg/m^2^. The most common grade 3 adverse events were febrile neutropenia, pneumonia, and thrombocytopenia. There were no differences in the PK between the regimens or between days in a particular regimen. As shown in Figure 4, SGI-110 was absorbed rapidly after subcutaneous injection. The half-life of SGI-110 was longer than that of decitabine. SGI-110 can be present in plasma for up to 8 h or more, resulting from the continuous appearance of decitabine, which creates an exposure window of 11–12 h. In summary, SGI-110, as a prodrug for decitabine, could release decitabine slowly and has a longer exposure window (Issa et al., 2015).

#### 3.2.2 RX-3117

RX-3117 (TV-1360, **6**) is a potent DNA synthesis inhibitor. A study showed that RX-3117 could inhibit the proliferation of breast, lung, and colon cancer cells with an IC_50_ of 0.18, 0.25, and 0.28 μM *in vitro*, respectively (Choi et al., 2012). RX-3117 induced 100%, 78%, 62%, and 66% tumor growth inhibition in human Colo 205, H460, H69, and CaSki tumor xenograft models *in vivo*, respectively. Further studies showed that RX-3117 could be phosphorylated to diphosphate (RX-DP) and triphosphate (RX-TP) by uridine cytidine kinase. RX-DP is reduced by ribonucleotide reductase to DRX-DP and further phosphorylated to DRX-TP, which can incorporate into a DNA sequence to inhibit DNA synthesis (Peters et al., 2013), while RX-TP can incorporate into RNA to inhibit RNA synthesis.

In a phase II clinical trial, patients with advanced urothelial cancer received a dose of 700 mg of RX-3117 orally once-a-day for 5 consecutive days followed by 2 days off per week for 3 weeks. Among the 45 patients with refractory metastatic pancreatic cancer, progression-free survival (PFS) increased by more than 2 months in 13 patients, disease stability increased by more than 4 months in 5 patients, and partial remission in 1 patient. The adverse effects were anemia, fatigue, and diarrhea. Patients with solid tumors were treated with 50 mg or 100 mg RX-3117 orally or 20 mg intravenously. As shown in Figure 4, RX-3117 has high oral bioavailability (F) and the absolute F was 55.67% and 33.42% for the 50 and 100 mg doses, respectively, which suggests that the F of RX-3117 in plasma may not be dose-proportional. However, the half-life of RX-3117 is proportional to the dose. The plasma PK of intravenous RX-3117 differed from that of oral RX-3117. The 20-mg dose of intravenous RX-3117 had a mean C_max_ of 1,143.63 ng/ml, which was approximately a fourfold increase over the peak concentrations of the oral doses. Therefore, a low dose of RX-3117 administered intravenously may be a good choice for the treatment of solid tumors (Udvaros et al., 2015).

#### 3.2.3 Pyrimidine derivant: 5-Fluoro-2′-deoxycytidine

In order to solve the instability of decitabine, one pyrimidine analog, 5-fluoro-2-deoxycytidine (FdCyd, **7**), was designed by adding fluorine to the pyrimidine ring (Frank and Robert, 2005). FdCyd inhibits DNA methylation by the same mechanism as decitabine. It can inhibit DNA methylation by integrating into DNA sequences in the form of triphosphate or binding to DNMT covalently.

FdCyd is currently in phase II clinical trials for the treatment of solid tumors. A total of 54 patients with advanced malignant tumor received intravenous infusion of FdCyd and tetrahydrouridine (THU, inhibiting the deamination of FdCyd) for 3 h for 5 days every 3 weeks (Newman et al., 2015). The dose of FdCyd was 2.5–180 mg/m^2^/day and the fixed dose of THU was 350 mg/m^2^/day. A total of 20 patients with advanced solid tumor had a stable disease plateau between 1.4 and 13.3 months and their CT results showed that the tumor volume reduction was more than 90%. Significant clinical improvement was noted after two cycles of treatment, the PR was obtained after four cycles, and continued shrinkage of the tumor was noted after six cycles. One of six patients at a dose of 134 mg/m^2^/day experienced a first-cycle DLT, grade 3 colitis. The FdCyd volume of distribution of 30–40 L/m^2^ suggests the distribution of FdCyd in body water, consistent with its hydrophilic character.

#### 3.2.4 Azacitidine derivant: 5, 6-Dihydro-5-azacitidine

5, 6-Dihydro-5-azacytidine (DHAC, **8**) was designed by opening the imine bond at the C5-C6 position of azacitidine. However, DHAC did not show good efficacy in the treatment of mesothelioma either as a single agent or in combination with cisplatin and the response in solid tumors was limited; therefore, the clinical trial had to be terminated (Pechalrieu et al., 2017).

A total of 13 patients with advanced cancer were administered by infusion over 24 h with a dose of 1,000–7,000 mg/m^2^ (Curt et al., 1985). Dramatic reductions in palpable adenopathy were observed in two patients. The limiting toxicity was pleuritic chest pain. As shown in Figure 4, DHAC was absorbed slowly with a T_max_ up to 8 h. A total body clearance of 311 ± 76 ml/min/m^2^ and post-infusion half-life during 1 and 2 hours were observed. 40% DHAC was excreted unchanged in urine. The slower clearance rate and the large quantity recoverable in the urine are most likely attributable to the chemical stability. It is worth noting that DHAC is stable in aqueous solution and may be administered intravenously, which potentially avoids the acute toxicities associated with the bolus administration of 5-azacytidine.

#### 3.2.5 Ara-C and azacitidine derivant: Fazarabine

Fazarabine (**9**) was synthesized in the 1970s as a molecule with the arabinose of ara-C and the triazine ring of azacitidine (Beisler et al., 1979). Like ara-C, fazarabine requires intracellular activation ofthe nucleotide triphosphate by deoxycytidine kinase before integrating into a DNA sequence. It is reported to be cytotoxic against the Molt-4 human lymphoblastic leukemia cell *in vitro* (Townsend et al., 1985). In addition, fazarabine has a better activity against both murine and human solid tumor in contrast to its parent compounds (ara-C and 5-AC), including Lewis lung carcinoma and TE-671 medulloblastoma (Dalal et al., 1986).

Fazarabine has undergone several phase I/II clinical trials for the treatment of cancer, which has been discontinued due to low response rates. A total of 16 children with refractory malignancies were administered fazarabine as a 24-hour continuous infusion with a dose of 15 or 20 mg/m^2^/h (Heideman et al., 1989). No objective tumor responses were noted. Two patients had stable disease for a period of 6 weeks. DLT was granulocytopenia. As shown in Figure 4, there was a rapid increase in plasma concentrations of fazarabine after infusion. Fazarabine is widely distributed and it can cross over the blood–brain barrier into the cerebrospinal fluid. However, these experiments failed to correct the considerable spontaneous degradation of fazarabine in aqueous media (half-life, 3.9 ± 0.45 h in RPMI 1640 medium). Thus, it is possible that the plasma concentrations maintained during continuous infusion may be even more cytotoxic than that was observed.

#### 3.2.6 Adenosine derivants: Cladribine and fludarabine

Adenosine analogs DNMTi cladribine (**10**) and fludarabine (**11**) are mainly used for the treatment of hematologic tumors in clinics. They can inhibit DNA methylation through various mechanisms. First, they can be integrated into DNA sequences in the form of triphosphate deoxynucleotide to inhibit DNA synthesis like 5-azacytidine. Second, similar to clofarabine, they blocked the PCNA protein to promote the activity of DNMT1 on semi-methylated DNA by upregulating p21 (Iida et al., 2010). Third, they can also inhibit the activity of SAH hydrolase, a key enzyme for methyl donor recycling, thus decreasing the level of both DNA and histone methylation (Wyczechowska and Fabianowska-Majewska, 2003). It is worth noting that fludarabine as a single drug has no effect on the methylation of histone H3 in AML cells, but can promote the methylation of multiple sites of histone H3 when combined with clofarabine, which is a very interesting question and deserves further study (Valdez et al., 2011).

Cladribine is a nucleoside analog of deoxyadenosine. A chlorine substitution in the purine ring protects cladribine against degradation by adenosine deaminase. Cladribine is currently used for the treatment of multiple sclerosis (MS). The recommended dose is 3.5 mg/kg, consisting of two annual courses of treatment, each comprising two treatment weeks every other month. There were 1,326 patients with MS who received either placebo or a cumulative dose of cladribine of 3.5 or 5.25 mg/kg over the 2-year study period for two courses of treatment. Results showed 86% relative reduction in the mean number of T1 Gd + lesions, 73% relative reduction in the mean number of active T2 lesions, and 74% relative reduction in the mean number of combined unique lesions per patient per scan. As shown in Figure 4, patients with MS were treated with a single dose of 10 mg cladribine tablets by month. Cladribine was rapidly absorbed. Cladribine has good oral bioavailability of about 40%, and it has the potential to penetrate the blood–brain barrier. Cladribine is phosphorylated to cladribine monophosphate (Cd-AMP) by deoxycytidine kinase in cells. Cd-AMP is further phosphorylated to chlorodeoxyadenosine diphosphate (Cd-ADP), which is in turn phosphorylated by 5-nucleotidase to Cd-ATP. Cladribine elimination is equally dependent on renal and non-renal routes (Merck Serono Europe Limited, 2017).

Fludarabine is a fluorinated derivative of adenosine, which can prevent the deamination of adenosine deaminase. Patients with B-cell CML received 10 mg tablets of fludarabine to a dose of 40 mg/m^2^/d for 5 days. The OR was 71.6% (CR, 37.0%; PR, 34.6%). The median time to progression was 841 days. The grade 3/4 toxicity was myelosuppression (Rossi et al., 2004). In another study, 27 patients with non-Hodgkin’s lymphoma and B-cell CML received fludarabine orally and intravenously. As shown in Figure 4, fludarabine was rapidly absorbed within 1 h of oral administration. AUC_0–24h_ increased in a linear proportion to the oral dose, which is the same as intravenous administration. Bioavailability and time to C_max_ were dose-independent. There was no significant difference in the PK between oral fludarabine with or without food. Taken together, oral doses of fludarabine can achieve an AUC_0–24h_ of 2-fluoro-arabinofuranosyl-adenine similar to intravenous administration with dose-independent bioavailability. Fludarabine is largely eliminated by renal excretion.

#### 3.2.7 Anesthetic agent: Procaine

Procaine (**12**) has been used as an anesthetic for years, and it was not known that procainamide could inhibit DNA methylation until 1988 (Cornacchia et al., 1988). Inspired by this, researchers turned to procaine, which has a similar structure to procainamide. Procaine (10 nM) inhibited the proliferation of MCF-7 breast cancer cells and reduced the methylation of TSG RARβ by 40% (Villar-Garea et al., 2003). Studies showed that procaine reduced the affinity between DNMT1 and semi-methylated DNA or DNMT1 and SAM by binding to the CpG of DNA at a high concentration of 100–500 μM (Lee et al., 2005). Procaine is easily absorbed from the injection site and distributed to systemic tissues. It can pass the blood–brain barrier and placenta and also can be hydrolyzed by esterase in tissues and plasma. Most of the hydrolyzed products are excreted through urine and a small amount is metabolized by the liver. These data indicate that procaine is a drug with limited distribution and tissue uptake, as well as a short duration of action. As an infiltration anesthetic, 0.25%–0.5% solution can be used in the department of stomatology, no more than 1 g at a time.

#### 3.2.8 Polyphenolic compound: Epigallocatechin gallate

Epgallocatechin gallate (EGCG, **13**) is a polyphenolic compound extracted from green tea. Studies showed that EGCG inhibited the methylation of WIF-1 in lung cancer cells (Gao et al., 2009) and GSTP1 in prostate cancer cells (Pandey et al., 2010). In addition, EGCG could induce a decrease in the global DNA methylation level in the human epidermoid carcinoma A431 cell by reducing the mRNA and protein levels of DNMT1, DNMT3a, and DNMT3b, especially for TSGs, p16INK4a, and Cip1/p21 (Nandakumar et al., 2011). The mechanism of DNA methylation inhibition is manifested in the following two aspects. First, EGCG inhibited DNMT1 by binding to the catalytic site of DNMT1 non-covalently with a Ki of 6.89 μM (Fang et al., 2007). Second, catechol-O-methyltransferase introduces the methyl of SAM to the catecholamine of EGCG, which leads to depletion of SAM available as a methyl donor, thereby causing an indirect reduction in DNMT-mediated DNA methylation (Hong et al., 2003). In addition to inhibiting DNA methylation, EGCG can also exert anti-tumor activity by regulating the methylation and acetylation of histones. For example, EGCG could promote the transcription of human telomerase reverse-transcriptase (hTERT) by decreasing the acetylation of lysine on histone H3 and H4 in breast cancer cells (Meeran et al., 2011), while increasing the acetylation of lysine 9/14 on histone H3 and lysine 5/12/16 on histone H4 in skin cancer A431 cells (Nandakumar et al., 2011). In addition, EGCG could also inhibit the tri-methylation of lysine 27 on histone H3 and increase the acetylation of lysine 9/18 on histone H3 at the TIMP-3 promoter in prostate cancer cells (Deb et al., 2019).

A total of 40 volunteers received a formulation of 800 mg EGCG once daily, a formulation of 400 mg EGCG twice daily, or placebo once daily. There was no significant difference in the PK between the two doses, while C_max_ and AUC increased in a dose-dependent pattern. As shown in Figure 4, EGCG was absorbed slowly and distributed widely in the body, which was first absorbed in the intestine. However, the bioavailability is limited due to its oxidation and metabolism (Chow et al., 2003).

#### 3.2.9 Muscle relaxant: Hydralazine

Hydralazine (**14**) is a smooth muscle relaxant used to treat hypertension. Inspired by the ability to induce reduced DNA methylation in systemic lupus erythematous cells, hydralazine was identified as a DNMTi. A study showed that hydralazine induced demethylation and re-expression of TSG p16 and RAR2 in human bladder cancer and APC in human cervical cancer HeLa and Caski cells (Leung et al., 2011). The mechanism of hypomethylation of hydralazine was controversial. First, docking results showed that hydralazine could bind to DNMT1 as a weak DNMTi. Second, hydralazine decreased the expression of human DNMT1 and DNMT3A. In addition, hydralazine may not only affect DNA methylation but can also be involved in the negative regulation of methylation of lysine 9 on histone H3. Studies have shown that hydralazine could inhibit the dimethylation of lysine 9 on histone H3 by inhibiting the activity of histone methyltransferase G9A in gemcitabine-resistance cervical cancer cells, thereby upregulating the expression of the human equilibrative nucleoside transporter 1 (hENT1) and reversing drug resistance. This has also been demonstrated in enzyme inhibition experiments; hydralazine at 2 μM and 10 µM strongly reduced the methylation of lysine 9 on histone H3 (Candelaria et al., 2012).

In a phase II clinical trial, 14 patients with progressive cutaneous T-cell lymphoma received hydralazine plus magnesium valproate. The OR was 71% (CR, 50%; PR, 21%). The median time to response was 2 months, the median duration of response was 28 months, and the median PFS was 36 months (Candelaria et al., 2007). Healthy volunteers were given an oral administration of 100 mg hydrazine. The oral bioavailability is 25%–55% and half-life is about 2–8 h. An obvious effect was noted 1 hour after oral administration and lasted for 24–30 h. It is mainly metabolized in the liver, mostly excreted in the form of acetylated and hydroxylated metabolites, of which 3%–14% is excreted through urine in its original form (Ludden, 1982).

#### 3.2.10 Flavonoid derivants: Genistein and equol

Genistein (**15**) and equol (**16**) are flavonoid compounds extracted from legumes. Equol induced the promoter hypomethylation of TSGs BRCA1 and BRCA2 in breast cancer cells (Bosviel et al., 2012). Genistein can reverse the hypermethylation and re-expression of the P16, RARβ2, MGMT, PTEN, and CYLD genes in esophageal and prostate cancer cells (Fang et al., 2005). Enzyme activity results showed that genistein could inhibit the activity of DNMT1 and DNMT3 (Majid et al., 2010). In addition, genistein could also regulate the epigenetics of histones. A study showed that genistein could significantly reduce the methylation of lysine 9 on histone H3 and decrease both the transcriptional and translational levels of HDAC2 in a patient-derived xenograft (PDX) orthotopic mouse TNBC model (Sharma et al., 2021). Equol, like genistein, could significantly reduce the methylation of lysine 4/27 on histone H3 in MCF-7 and MDA-MB 231 breast cancer cells, while significantly increasing the acetylation of lysine 4/8 on histone H3 (Dagdemir et al., 2013).

In a phase I/II study, 13 patients with metastatic colorectal cancer were treated with genistein plus FOLFOX. The adverse events related to genistein alone were headaches, nausea, and hot flashes. The OR and PFS were 61.5% and 11.5 months, respectively. As shown in Figure 4, 40 volunteers received a dose of 30, 60, 150, or 300 mg genistein orally. Genistein was rapidly absorbed and showed nearly dose-linear PK characteristics. The plasma concentration–time profiles showed a rapid one-peak course until T_max_, followed by a multiphasic decrease, which probably reflected a distribution and elimination phase. Genistein was safe and well-tolerated in the dose range of 30–300 mg. ADME studies revealed that intestinal, biliary, and renal excretions are the excretion pathways for genistein (Ullmann et al., 2005).

Equol has been shown to reduce the risk of prostate cancer in men and breast cancer in women. The incidence of breast cancer in 159 patients was reduced by 20% after intervention with soy isoflavones. The PK of equol was assessed in 14 volunteers by oral administration of 20 mg. The plasma concentration reached the maximum within 1–2 h and decreased rapidly in the first 6 h, showing a unimodal distribution. The half-life is 7–8 h. 61% of the dose was excreted through urine in 72 h. The high bioavailability of equol suggests that low doses of equol taken twice daily may be sufficient (Setchell et al., 2009).

#### 3.2.11 Diketone derivant: Curcumin

Curcumin (**17**) is a kind of diketone compound extracted from turmeric, which has anti-inflammatory, anti-oxidant, anti-tumor, anti-angiogenesis, anti-bacterial, anti-viral, anti-fibrotic, anti-depressant, anti-ischemic, immunomodulatory, and neuroprotective properties. The anti-tumor activity of curcumin is mainly attributed to its anti-inflammatory and anti-oxidant properties. Curcumin has also been reported to exert anti-tumor activity by modulating growth factors, enzymes, transcription factors, and kinase- and apoptosis-related proteins (Giordano and Tommonaro, 2019). In addition, the anti-tumor activity of curcumin is also associated with epigenetic modifications, primarily DNA methylation. Curcumin was identified as a DNMTi through virtual screening. Molecular docking results suggested that curcumin blocked the catalytic thiolate of C1226 of DNMT1 covalently, which was validated by the fact that curcumin inhibited the activity of DNMT (M. SssI) with an IC_50_ of 30 nM (Liu et al., 2009a). Curcumin can inhibit the DNA methylation of RAR2 and FANCF in cervical cancer cells *in vitro* (Parashar et al., 2012). A 70% decrease in tumor growth in a xenografted MV4-11 model was achieved when the mice were treated with 100 mg/kg curcumin *in vivo*. In addition to inhibiting DNA methylation, curcumin could also inhibit the methylation of histones. For example, curcumin inhibits the methylation of lysine 4/9/27 on histone H3 in leukemia cells by inhibiting histone lysine methyltransferase (HKMT) and enhancing the activity of histone lysine demethylase (HKDMT) with consequent changes in gene expression that could contribute to its antitumor effect. Interestingly, curcumin also reduced the methylation of arginine 42 and glutamine 19/55 on histone H3 (Sawesi et al., 2022).

In a phase I clinical trial, 32 patients with metastatic cancer were treated with liposomal curcumin infusions at doses between 100 and 300 mg/m^2^. Of 15 patients under tumor assessment, 14 showed progressive disease, one showed stable disease, and two had significant tumor marker responses. No DLT was observed in 26 patients at doses between 100 and 300 mg/m^2^ over 8 h. The adverse events were facial edema, anemia, and hemolysis. PK analyses showed that there is a stable plasma concentration of total curcumin during infusion and a rapid decline after the infusion as a consequence of redistribution and extensive metabolism. Plasma concentrations of curcumin showed an apparent linear dependence on the infusion rate. Curcumin has low oral bioavailability in humans and it may undergo intestinal metabolism (Sharma et al., 2001).

#### 3.2.12 Alcohol abuse drug: Disulfiram

Disulfiram (**18**), a clinical drug for the treatment of alcohol abuse, was found to be a DNMTi by chance. Disulfiram has a sulfhydryl group, which can attack the sulfhydryl of DMNT cysteine to inhibit the activity of DMNT. A study showed disulfiram could inhibit DMNT1 and induce the expression of TSG, such as, p16, and RARβ2 (Lin et al., 2011a). In addition, disulfiram could also decrease the dimethylation and trimethylation of lysine 4 on histone H3 by inducing the degradation of histone methyltransferases MLL1 and MLL2 in pediatric glioma cells (Meier et al., 2021).

Disulfiram is currently in phase II clinical trials for cancer treatment. The efficacy in the treatment of prostate cancer was tested in 19 patients; two cohorts received disulfiram 250 mg and 500 mg daily, respectively (Schweizer et al., 2013). Changes in the global 5^me^C content were observed in 2 of 9 patients (22.2%) in cohort 1 and 3 of 10 (30.0%) in cohort 2. Six patients developed grade 3 AEs. As shown in Figure 4, the half-life was 7.3 h and the average peak time was 8–10 h when the dose was 250 mg. However, the plasma concentrations varied among individuals, which might be caused by the strong lipid solubility and the hepatointestinal circulation. Disulfiram is absorbed primarily from the gastrointestinal tract and is quickly reduced by glutathione in red blood cells to its monomer. Most of the metabolites are excreted through urine, while carbon disulfide is excreted through the respiratory system.

#### 3.2.13 Anthraquinone terpenoid compound: Resveratrol

Resveratrol (**19**) is an anthraquinone terpenoid found in peanuts with cardiovascular protection effect. The demethylation activity of resveratrol has been shown to be lower than that of EGCG, while it has synergistic effects in combination with cladribine or fludarabine. The mechanism of demethylation lies in the fact that it can reduce the expression of DNMT1 and inhibit the acetylation of STAT3, which can destroy the interaction between acetylated STAT3 and DNMT, thus inhibiting CpG methylation (Lee et al., 2012). Resveratrol can also catalyze post-translational modifications of histones. A study showed that resveratrol decreased the dimethylation of arginine 3 on histone H4 and the trimethylation of lysine 27 on histone H3 by decreasing the expression of arginine methyltransferase 5 in breast cancer cells. It could also enhance the acetylation of lysine 9/27 on histone H3 by inhibiting the activity of lysine deacetylase. These epigenetic modifications lead the expression of genes BRCA1, p53, and p21 and inhibit the growth of breast cancer cells (Chatterjee et al., 2019).

A total of 24 patients with relapsed or refractory multiple myeloma were enrolled in a phase II clinical trial. Nine patients had SD following a minimum of two cycles, and nine patients received a resveratrol and bortezomib combination, achieving an ORR (≥MR) of 22% by a modified intention-to-treat (ITT) population, or an ORR of 8% by ITT analysis. The median time to progression was 2.8 months and overall survival was not reached. Adverse events were nausea, diarrhea, and vomiting. A total of 10 volunteers received a dose of 0.5, 1, 2.5, or 5 g resveratrol orally (Boocock et al., 2007). As shown in Figure 4, resveratrol was rapidly absorbed, reaching a maximum plasma concentration of 0.83–1.5 h, followed by a sharp decline, which was distributed in the organs with abundant blood perfusion. Plasma concentrations showed a “bimodal shape” after oral administration, possibly due to enterohepatic recirculation. AUC and C_max_ for resveratrol increased with dose. Resveratrol has low bioavailability, which was consistent with the whole body clearance (2,235–4,930 L/h) and volume of distribution (9,198–22,226 L). Resveratrol was metabolized to glucuronylated and sulfated resveratrol rapidly.

#### 3.2.14 Phenolic acid: Caffeic acid

Caffeic acid (**20**) is a phenolic acid compound derived from honeysuckle, which is often used in the prevention and treatment of bleeding. As a weak DNMTi, it could inhibit DNMT from Spiroplasma sp. strain MQ1 (M Sssl) and human DNMT1 with an IC_50_ of 3.0 mM and 2.3 mM, respectively. Further study showed that caffeic acid inhibited DNA methylation in a non-competitive way. Caffeic acid, as a substrate of catechol-O-methyltransferase, is methylated to SAH, which further inhibits DNMT1 (Zhu and Zhu, 2006). Dilshara et al. (2016) showed that 50 μM of caffeic acid increased the apoptosis of hepatocarcinoma Hep3B cells *in vitro*. Caffeic acid (100 mg/kg) can reduce the changes in markers of liver injury in hepatocarcinoma in rats. In addition, caffeic acid could also inhibit the activity of HDAC with an IC_50_ of 2.54 mM. Its activity is not as potent as we expected, whereas its derivatives such as curcumin and chlorogenic acid have potent activity for HDAC inhibition, which suggests that caffeic acid may be considered a promising lead structure for HDAC inhibitors that have the potential to provide new therapeutics for cancer (Bora-Tatar et al., 2009).

Caffeic acid is in phase II clinical trials for cancer treatment. As shown in Figure 4, the maximum plasma concentration of caffeic acid was observed 1 h after ingestion of food and then rapidly decreased. Only a small amount of caffeic acid is absorbed in the stomach, while 95% absorbed in the colon by the intestinal mucosa (de Oliveira and Markowicz Bastos, 2011). The absorbed caffeic acid converts to its metabolites through methylation, sulfation, and gluconic acid, which are excreted through urine finally.

### 3.3 DNA methyltransferase inhibitors in preclinical research

#### 3.3.1 Nucleoside DNMTi

Although three nucleoside DNMTis azacitidine, decitabine, and clofarabine are already on the market, they are chemically and metabolically unstable with low bioavailability. Thus, a large number of stable second-generation nucleoside DNMTis were developed, which include azacitidine derivatives CP-4200 and zebularine, decitabine derivatives NPEOC-DAC, and thio-cytidine derivatives T-dCyd and 5-aza-t-dCyd.

**FIGURE 5:**
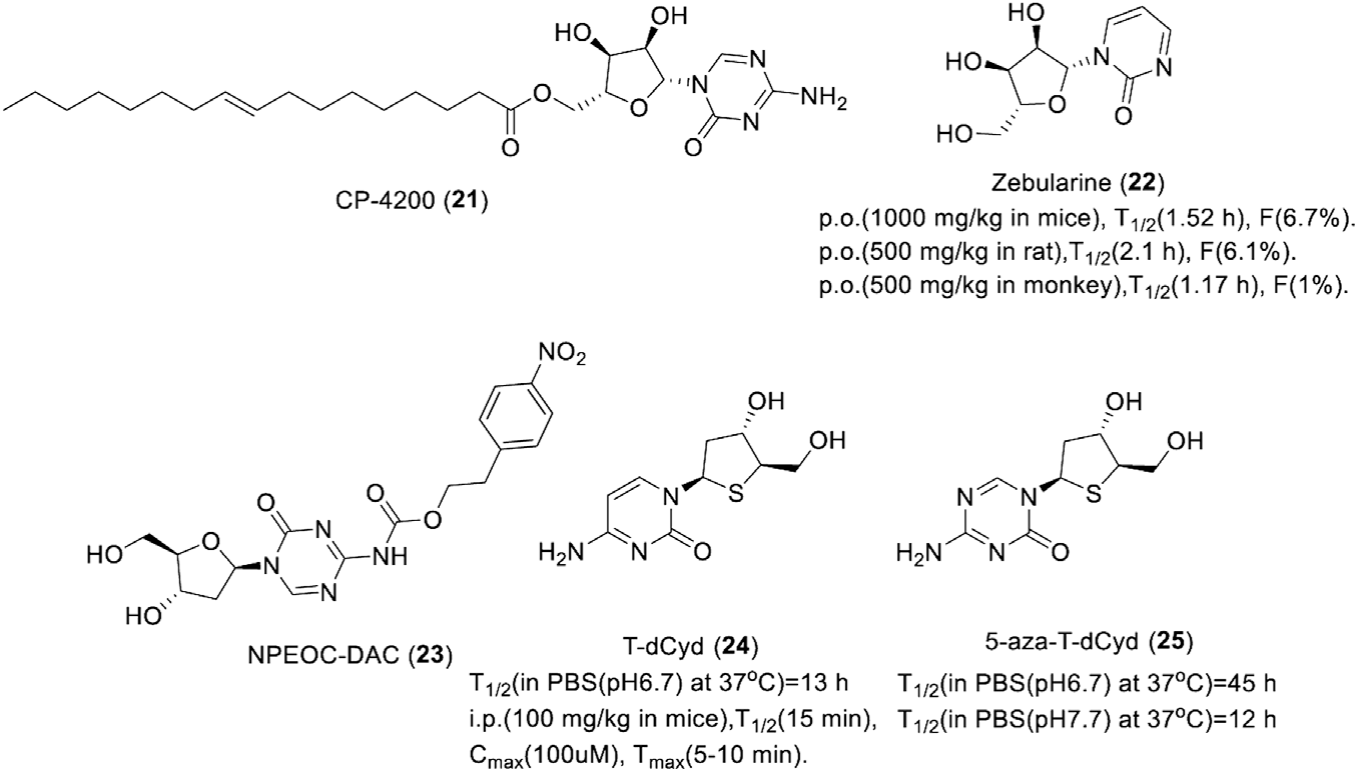
Chemical structure of nucleoside DNMTis in preclinical.

CP-4200 (**21**) is an elaidic acid ester of azacitidine that was synthesized with the intention of rendering drug uptake less dependent on the conventional nucleoside transport systems and improving the cellular uptake. As the trans-octadecenoate of azacitidine, the cellular uptake was substantially less dependent on the nucleoside transporters, and it has better membrane permeability. CP-4200 (Figure 5) has better anti-tumor activity than azacitidine in an orthotropic mouse AML model *in vivo* (Brueckner et al., 2010). As a prodrug of azacitidine, CP-4200 has the advantage of delayed delivery of azacitidine. It could inhibit the methylation of the whole genome and specific genes in human colon cancer HCT116 cell and breast cancer MCF7 cell effectively, which is dependent on the activity of DNMT1 degradation (Brueckner et al., 2010). CP-4200 is currently in the preclinical stage for cancer treatment.

Zebularine (**22**), an azacitidine derivative lacking an amino at the 4th position of the pyrimidine ring, exerts demethylation activity by stabilizing the binding of DNMT to DNA. A study showed that it could induce hypomethylation in the promoter of TSG p57 and p15 in myeloid leukemia cells *in vitro* (Nakamura et al., 2015). DNMT1 was almost completely suppressed in tumor cells by zebularine, indicating it has high selectivity toward DNMT1. Zebularine could lead the promoter demethylation of p15INK4B and induce p15INK4B expression in AML194 cells by maintaining the high acetylation level of histone H4 and increased the trimethylation of lysine 4 on histone H3 when combined with trans-retinoic acid or phenyl sodium butyrate but had no effect on histone modification, especially histone methylation, when used as a single drug (Scott et al., 2007). As show in Figure 5, Zebularine could be continuously administered to maintain the demethylated state due to its low toxicity. It also works when given orally. However, due to saturation of the absorption process and primary metabolism of the liver, zebularine has limited oral bioavailability. Plasma concentrations declined with a short half-life in mice, rats, and monkeys, which indicates that frequent dosing or continuous i.v. infusion may be necessary to maintain the prolonged inhibition of DNMT.

NPEOC-DAC (**23**, Figure 5) is a decitabine derivative formed with the introduction of a 2′-deoxy-N4-[2-(4-nitrophenyl)ethoxycarbonyl] (NPEOC) group in the 4-amino of decitabine, which could be cleaved by carboxyl esterase to release decitabine (University of Southern California, 2008). NPEOC-DAC inhibits global DNA methylation by the induction of hypomethylation of the LINE-1 repetitive element and it reverses the hypermethylation of TSG ID4 in tumor cells. The ability to inhibit the DNA methylation of NPEOC-DAC is specific to HepG2 and Hep3B2 liver cancer cell lines, which is dependent on the activity of carboxyl esterase 1 (Byun et al., 2008). NPEOC-DAC was 23-fold less efficient with a 3-day delay at inhibiting DNA methylation than decitabine, which suggested that NPEOC-DAC is capable of releasing decitabine slowly to maintain DNA hypomethylation by continuous administration. In addition, NPEOC-DAC has very low solubility in water, which could potentially lead to very favorable human PK compared to decitabine.

4′-Thio-2′-deoxycytidine (T-dCyd, **24**) and 5-aza-4′-thio-2′-deoxycytidine (aza-T-dCyd, **25**) are thiocytidine derivatives developed by the Southern Research Institute of the United States (Southern Research Institute, 2009). Studies showed that T-dCyd and aza-T-dCyd could deplete DNMT1 in cancer cells resulting from the re-expression of TSG p15. Both compounds depleted DNMT1 and inhibited tumor growth in human tumor xenograft models. Specifically, CCRF-CEM and KG1a leukemia cells were sensitive to both of them with an IC_50_ below 1 μM, while aza-T-dCyd was 3–15-fold more potent than T-dCyd. Aza-T-dCyd showed potential cytotoxicity toward NCI-H23 lung cancer cells, HCT-116 colon cancer cells, and IGROV-1 ovarian cancer cells with an IC_50_ of 4.5, 58, and 36 μM, respectively, while T-dCyd inhibited the proliferation of these cells at 100 μM by less than 50% (Thottassery et al., 2014). The selectivity of aza-T-dCyd was at least 10-fold more potent than that of decitabine. In addition, T-dCyd can incorporate into a DNA sequence with a high level and the presence of the thio-deoxyribose appears to increase the chemical stability of 5-aza-T-dCyd when incubated in PBS solution. The half-life of T-dCyd and aza-T-dCyd was 13 ± 1 h and 45 ± 6 h in PBS (pH 6.7) at 37°C (Figure 5), respectively.

#### 3.3.2 Non-nucleoside DNMTi

##### 3.3.2.1 Indole-based DNMTi

RG108 (**26**) is the first DNMT1 inhibitor discovered by rational drug design, which inhibits DNMT1 by blocking the active site of DNMT1 with an IC_50_ of 118 nM (Stresemann and Lyko, 2008). In a patent, 22 of 66 RG108 derivatives could inhibit DNA methylation with an IC_50_ of 50 μM or less (Cancer Research Technology Ltd, 2007). The modification of histones in tumor cells by RG108 is the same as that of zebularine.

**FIGURE 6:**
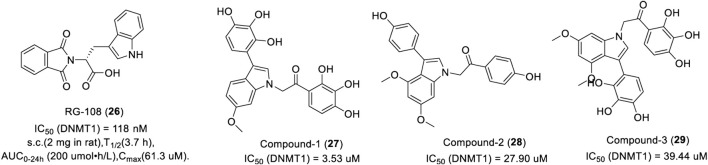
Chemical structure of indole-based DNMTis in preclinical.

As show in Figure 6, RG108 was slowly absorbed within 30 min of subcutaneous injection in rats and was widely distributed in various tissues, such as the liver, heart, and muscle. It takes about 4 h after absorption for the plasma concentration to decline by half, which indicates that RG108 has a long plasma half-life. In another study, researchers synthesized a large number of indole derivatives and evaluated the inhibitory activity against DNMT1 using tritium-labeled AdoMet (3H-SAM) as a methyl donor. Results showed that 17 of 27 indole derivatives could inhibit DNMT1 with an IC_50_ of 50 μM or less. Compound 1 (**27**) had the most potent inhibitory activity with an IC_50_ of 3.53 μM. Interestingly, compound 2 (**28**) exerted a more potent inhibition activity in CCFR-CEM hematologic cells and Jurkat over other cancer cell lines with an EC_50_ of 6.5 μM and 8.5 μM, respectively. Compound 3 (**29**) displayed a promising inhibitory effect in various cancer cells, representing a potential lead for further optimization (Ikerchem S L, 2014).

##### 3.3.2.2 Carbazole-based DNMTi

**FIGURE 7:**
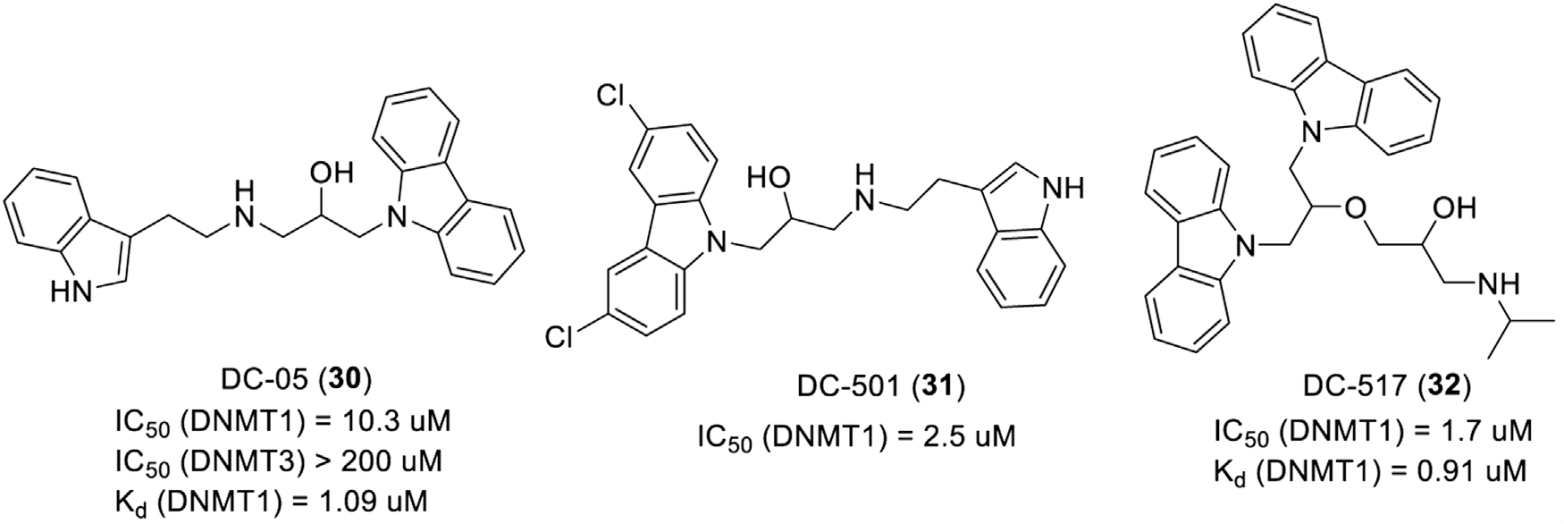
Chemical structure of carbazole-based DNMTis in pre-clinical.

Based on the crystal structure of mouse DNMT1 in complex with SAH and cytosine (PDB: 4DA4), a new carbazole DNMTi DC-05 (**30**) was found by a structure-based drug design. DC-05 is a selective DNMT1 inhibitor with an IC_50_ and Kd of 10.3 μM and 1.09 μM (Figure 7), respectively, which showed poor activities against DNMT3A/3B (IC_50_ > 200 μM). Due to the high IC_50_ and good selectivity of compound DC-05 to DNMT1, many DC-05 analogs were searched based on the structure of DC-05. DC-501 (IC_50_ = 2.5 µM, **31**) and DC-517 (IC_50_ = 1.7 µM, **32**) displayed 4.1- and 6.0-fold higher inhibitory activities against DNMT1 than that of DC-05 (Figure 7). The Kd of DC-517 is 0.91 μM, which suggested that DC-517 had a higher affinity for DNMT1. Further studies showed that DC-05, DC-501, and DC-517 can inhibit the proliferation of HCT116 and Capan-1 at a low micromolar concentration (Chen et al., 2014). The SAR analysis and predicted binding model study showed a better occupation of the cytosine and SAM pockets, and the formation of ionic interactions or hydrogen bonds linking the two pockets is necessary for the activity of carbazole DNMTi.

##### 3.3.2.3 Quinolone- and quinazoline-based DNMTi

**FIGURE 8:**
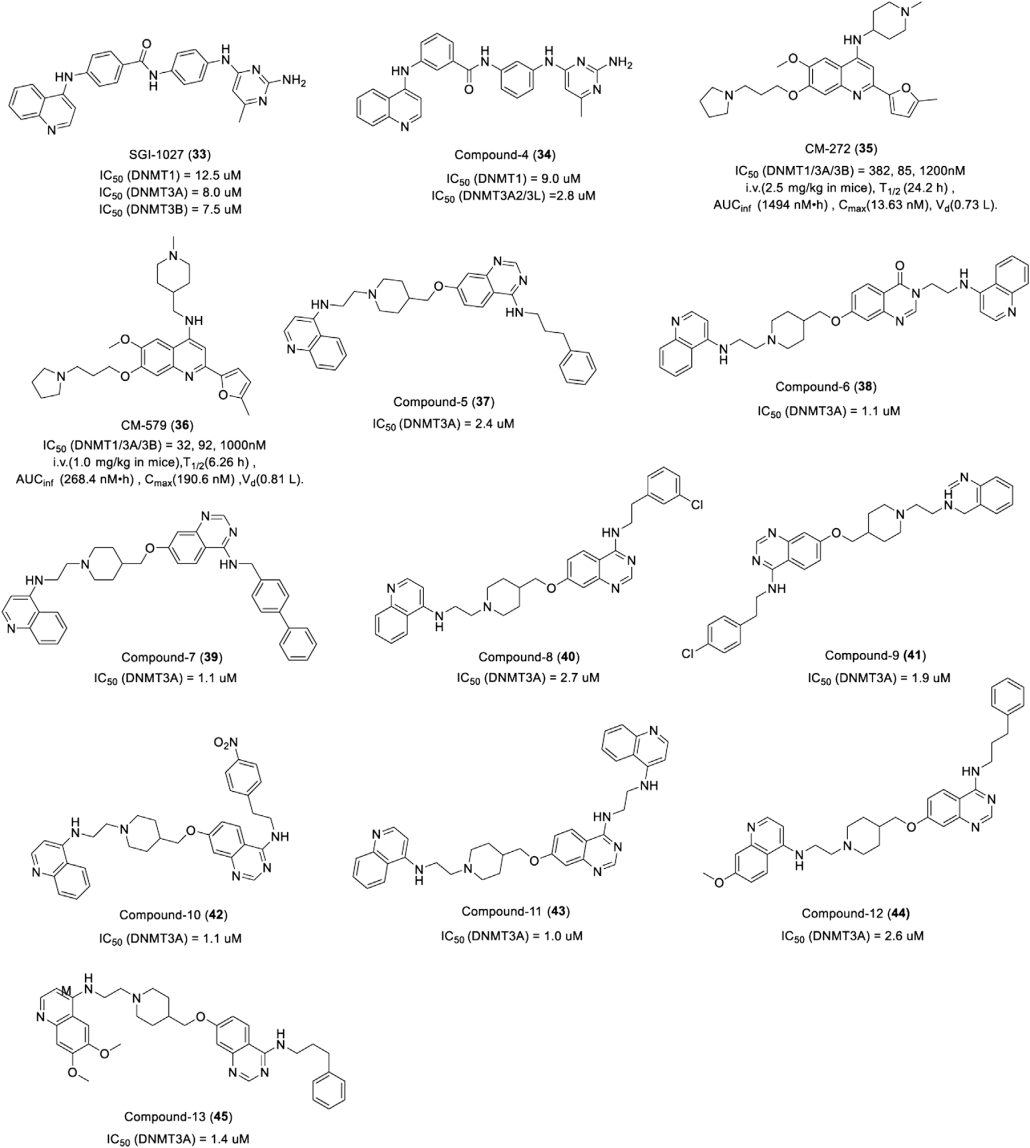
Chemical structure of quinolone- and quinazoline-based DNMTis in pre-clinical.

SGI-1027 (**33**) is a quinazoline DNMTi identified by SuperGen from 4-aniline quinazoline derivants, which showed potent activities against DNMT1, DNMT3A, and DNMT3B by a SAM-competitive mechanism with an IC_50_ of 12.5 μM, 8.0 μM, and 7.5 μM, respectively (Figure 8) (Datta et al., 2009). In addition, SGI-1027 interacted with DNA and resulted in SAM-noncompetitive but DNA-competitive inhibition. Treatment of human colon cancer RKO cells with SGI-1027 led to the re-expression of silenced TSG and the degradation of DNMT1. Furthermore, researchers prepared a series of regioisomers of SGI-1027 by shifting each fragment linkage from the para to the meta or ortho position, of which a novel quinolone DNMTi compound 4 (**34**) was identified (Figure 8). It was more potent than SGI-1027 and more selective toward other SAM-dependent methyltransferases with less toxicity (Valente et al., 2014).

CM-272 (**35**) and CM-579 (**36**) are other dual-target quinolone inhibitors targeting G9a and DNMT based on a rational drug design. CM-272 inhibits G9a, DNMT1, DNMT3A, and DNMT3B with an IC_50_ of 8 nM, 382 nM, 85 nM, and 1,200 nM, respectively (Figure 8), and promotes the apoptosis of various tumor cells *in vitro*. As an inhibitor of histone methyltransferase G9a, studies have shown that CM-272 can effectively reduce the di-methylation level of lysine 9 on histone H3 in hepatoma cells by inhibiting G9a and downregulating G9a expression (Bárcena‐Varela et al., 2019). CM-272 prolongs the survival of CEMO-1 cell xenogeneic models at a dose of 2.5 mg/kg. CM-579 showed more potent inhibitory activity against G9a, DNMT1, DNMT3A, and DNMT3B with an IC_50_ of 16 nM, 32 nM, 92 nM, and 1,000 nM (Figure 8), respectively, which has a high affinity for DNMT1 with a Kd of 1.5 nM. CM-272 and CM-579 have long plasma half-lives, among which the plasma half-life of CM-272 is four times than that of CM-579 (San José-Enériz et al., 2017). As show in Figure 8, Unfortunately, the distribution of both becomes narrow after absorption. Further studies found that the two compounds did not compete with SAM but inhibited the binding of G9a and DNMTs to their substrates reversibly, which may explain their selectivity and therapeutic potential in different tumors.

Quinazoline DNMTi was found in a series of quinazoline derivatives synthesized by Pierre Fabre Medicament. Among the 36 quinazoline derivatives, compounds 5, 6, 7, 8,9,10, 11, 12, and 13 (**37–45**) showed potent inhibitory activity against DNMT3A with an IC_50_ of 2.4, 1.1, 1.1, 2.7, 1.9, 1.1, 1.0, 2.6, and 1.4 μM, respectively (Figure 8). It is worth noting that compounds 6, 9, and 11 also had potent inhibitory activity against DNMT1 with an inhibitory rate of 100%, 75%, and 99% at 32 μM, respectively. Further study showed that compound 6 could inhibit the proliferation of human leukemia cell KG-1, lymphoma cell Karmas 299 with an EC_50_ of 0.5 and 1.8 μM (Figure 10), respectively. Compounds 6 and 7 could inhibit the proliferation of KG-1 with an EC_50_ of 1.3 and 0.4 μM, respectively (Pierre Fabre Medicament; Centre National de la Recherche Scientifique (CNRS), 2015).

##### 3.3.2.4 Bromotyrosine-based DNMTi

**FIGURE 9:**
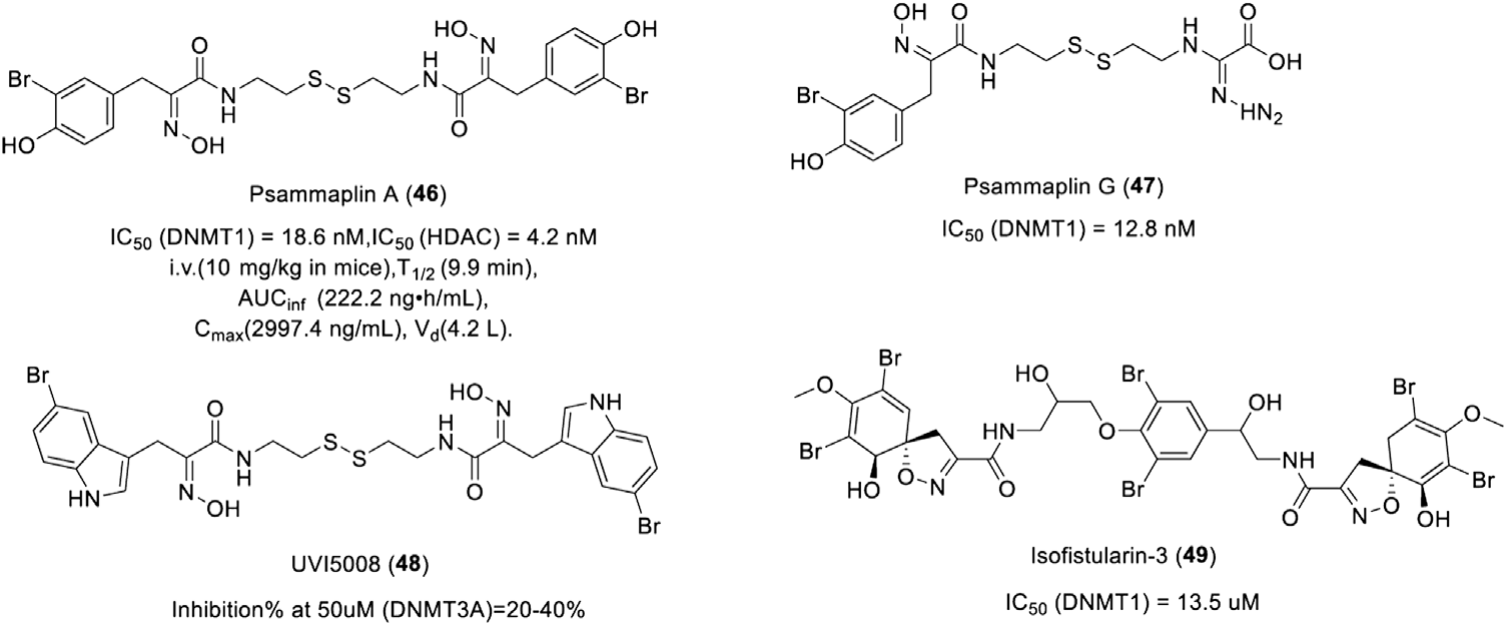
Chemical structure of bromotyrosine-based DNMTis in pre-clinical.

Psammaplin A (PsA, **46**) is a marine natural bromotyrosine compound extracted from the sponge *Psammaplinaplysilla*, which has inhibitory activity against HDAC and DNMT1 with an IC_50_ of 4.2 and 18.6 nM, respectively (Figure 9) (Piña et al., 2003). After intravenous administration of PsA in mice, the plasma concentration of PsA decreased rapidly with a plasma half-life of about 10 min. PsA was found to be highly distributed in lung tissues. The high and specific lung-targeting characteristics indicate that PsA has the potential to be developed as a lung cancer treatment agent. Psammaplin G (**47**), another bromotyrosine compound isolated from *Pseudoceratina purpurea*, exhibited inhibitory activity against DNMT1 with an IC_50_ of 12.8 nM (Figure 9) (Piña et al., 2003). UVI5008 (**48**), a derivative of Psammaplin A, could inhibit the DNA methylation of TSG p16^INK4a^ and RARβ2 (García et al., 2011), which showed strong anti-tumor activity with an IC_50_ from 0.2 to 3.1 µM. UVI5008 (Figure 9), a direct inhibitor of DNMT3a, has poor activity against DNMT1. To the best of our knowledge, UVI5008 is the first DNMT3a-selective inhibitor. In conclusion, UVI5008 was an epigenetic modifier that inhibited HDAC and DNMT, which efficiently induced the selective death of cancer cells and exerted its activity in a genetic mouse breast cancer model and several human tumor xenografts. As potent DNMT and HDAC inhibitors, Psammaplin A/G and UVI5008 can effectively inhibit DNA methylation and induce hyperacetylation of histone H3. Isofistularin-3 (**49**), a brominated alkaloid derived from aplysina aerophoba, was found to be a DNMTi due to its structural resemblance to psammaplin A. Isofistularin-3 could inhibit the activity of DNMT1 with an IC_50_ of 13.5 µM in a DNA-competitive manner (Figure 9). In addition, isofistularin-3 could modify the methylation of the AHR promoter in Raji cells and induce growth arrest of cancer cells in G0/G1 concomitant with increased p21 and p27 expression and reduced cycling E1, PCNA, and c-myc levels (Florean et al., 2016). These results suggest that bromotyrosine derivatives are potential DNMTis. However, a recent study found that Psammaplin A had no inhibitory activity against DNMT (García et al., 2011), and it was further confirmed that histone deacetylases are the main target of Psammaplin and not DNMT (Baud et al., 2012). The inhibitory activity and mechanism of these compounds on DNMT need to be further studied.

**FIGURE 10:**
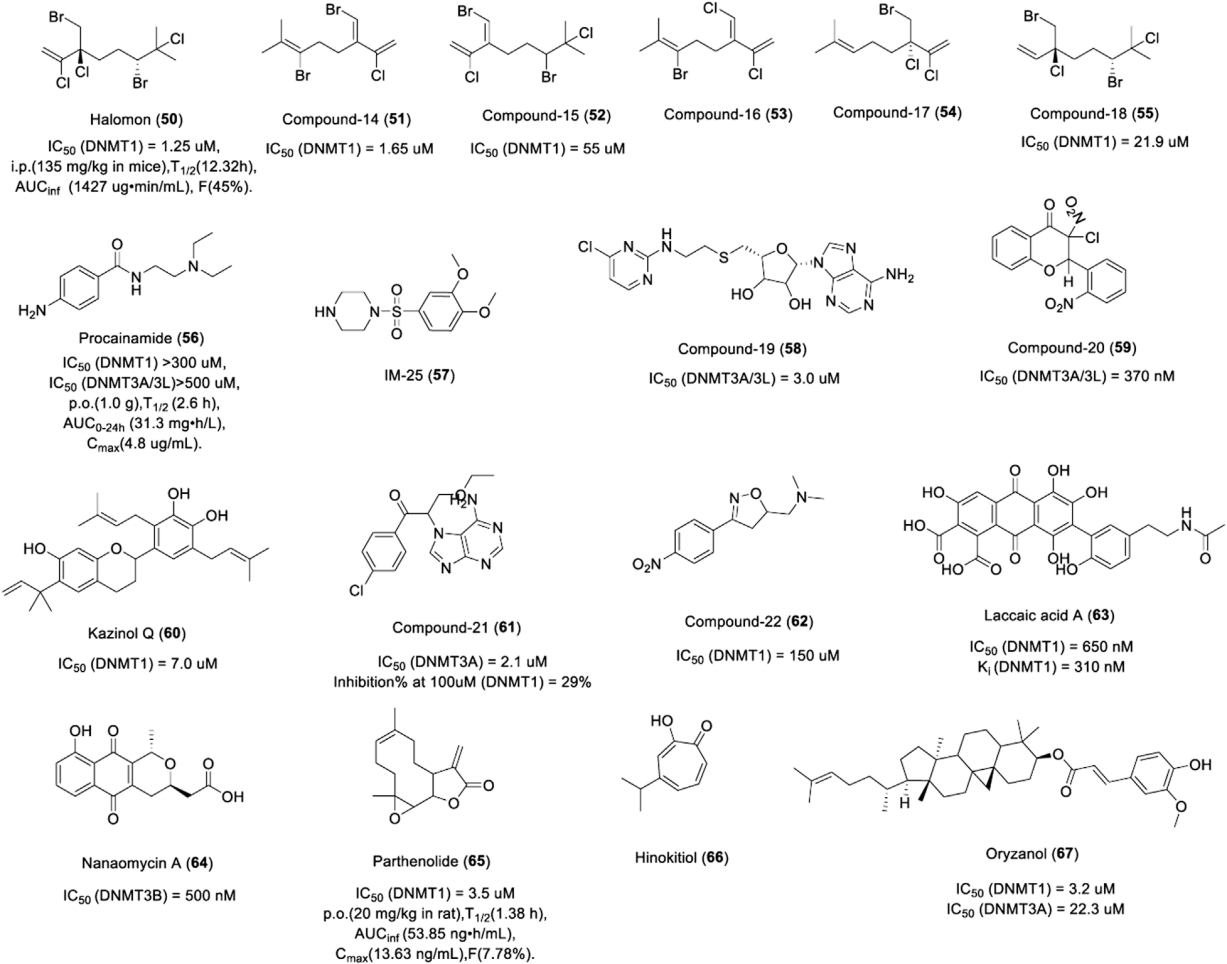
Chemical structure of other DNMTis in pre-clinical.

##### 3.3.2.5 Others

Halogenated monoterpenes, a class of marine products isolated from Madagascar red marine alga *Portieria hornemannii*, have been described as potential DNMTis (Andrianasolo et al., 2006). Previous studies have shown that halomon (**50**) and compound 14 (**51**) have comparable activities against DNMT1 (1.25 µM and 1.65 µM, respectively), while compounds 15 (**52**) and 18 (**55**) showed weaker inhibitory activity against DNMT1 with an IC_50_ of 55 µM and 21.9 µM (Figure 10), respectively. Compounds 16 (**53**) and 17 (**54**) were not tested due to the small quantities isolated. Halomon has a long half-life in mice. As show in Figure 10, The bioavailability of halomon was 45% and 47% after i.p and s.c administration, respectively. Urinary excretion of the parent compound was minimal. Halomon was distributed widely in all tissues.

Procaine, procainamide, and its derivant IM25 are aminobenzoic acid DNMTis (Figure 10). Procaine has entered the clinical stage of anti-tumor research while procainamide and IM25 are still in the preclinical research stage for tumor treatment. Procainamide (**56**) is a common antiarrhythmic drug, but studies have found that it also has the activity of inhibiting DNMT (Scheinbart et al., 1991). Studies showed that procainamide demethylated and re-expressed the RARβ2 and p16 genes in urinary bladder carcinoma and breast cancer cells (Cancer Research Technology Ltd, 2007) and the WIF-1 gene in lung cancer cells (Procaine et al., 2009). Further study showed that procainamide inhibited the activity of DNMT1 but not DNMT3A/3B (Chatterjee et al., 2019), suggesting that procainamide may be a selective inhibitor of DNMT1. Procainamide is almost completely absorbed after oral administration and has a long half-life; 50% of procainamide is metabolized to N-acetyl procainamide and excreted through the kidneys. IM-25 (**57**) is a novel amino benzoic acid DNMTi screened from 169 procainamide derivants by the Ohio State University, which caused the reversal of promoter hyper methylation of GSTP1 and TRIP10 genes in MCF7 breast cancer cells and induced global hypomethylation and hypomethylated more loci than procainamide and 5-aza-2′-deoxycytidine (Lin et al., 2011b). Procainamide and IM25 inhibited the activity of DNMT1 by reducing the affinity for its two substrates: SAM and hemi-methylated DNA, which is consistent with the inhibition mechanism of procaine. Although amino benzoic acids have been shown to be demethylating in a variety of tumor cells, their side effects limit their application in anti-tumor therapy.

Heteroaryl compounds are a new type of DNMTis produced by GlaxoSmithKline by imitating the structure of methyl donor SAM or SAH. These heteroaryl compounds contain a functional group, which could coordinate easily into the DNA binding region, particularly the cytosine binding pocket. Such small molecules can inhibit DNMT activity *via* binding in a covalent or non-covalent manner and, in turn, inhibit the enzymes. Of the five heteroaryl compounds synthesized in the first batch, compound 19 (**58**) has the most potent inhibitory activity against DNMT3A/3L with an IC_50_ of 3 μM (Figure 10) (Isakovic et al., 2009). A total of 120 heteroaryl compounds, synthesized in the second batch, exhibited an IC_50_ value in a range of 19.1–300 μM against DNMT1 and seven of them exhibited an IC_50_ value of less than 100 μM against DNMT1. The IC_50_ of these compounds for DNMT3B/3L was as low as 0.025 μM and as high as over 300 μM. Also, 24 compounds exhibited an IC_50_ value of less than 1 μM against DNMT3B/3L (Glaxosmithkline Intellectual Property Development Limited, 2013).

Based on the scaffold of genistein, 36 of 114 flavones and flavanone compounds were found as DNMT3 inhibitors, which could inhibit the murine DNMT3A/3L complex with IC_50_ values ranging from 0.37–315 μM. The most potent compound 20 (**59**) possesses a chlorinator motif at the C3 position of the flavanone ring with an IC_50_ of 370 nM (Figure 10). The docking results showed that it exerted inhibitory activity in a SAM-competitive manner (INSERM, 2011). In another study, researchers screened out another flavonoid DNMTi “Kazinol Q” (**60**) from 12 natural products, which exhibited potent inhibitory activity against DNMT1 by the same mechanism as EGCG with an IC_50_ of 7 μM (Figure 10) (Identification of Kazinol et al., 2014).

Propiophenone is a novel DNMT3A inhibitor, which was obtained by high-throughput screening in France. The best inhibitor was compound 21 (**61**), which showed potent inhibitory activity against DNMT3A with an IC_50_ of 2.1 μM, but showed weak activity against DNMT1 (29% at 100 μM) (Figure 10). The mechanism of the compound was investigated as it forms a reactive Michael acceptor group *in situ*. Another study showed that propiophenone derivatives are possibly transformed into Michael acceptors, which most likely inhibit DNMT3A by forming a covalent bond with the catalytic cysteine of the enzyme (Erdmann et al., 2015). Isoxazoline and oxazoline DNMTis are constrained analogs of procaine/procainamide by constraining the N-alkyl amide moiety into a 4-substituted- or 5-substituted-oxazoline ring. Results showed that compound 22 (**62**), an oxazoline DNMTi, had potent inhibitory activity against DNMT1 (IC_50_ = 150 μM) (Figure 10) and also exhibited a dose-dependent anti-proliferative effect against HCT116 human colon carcinoma cells. The docking results showed that compound 7b had the same binding site as SAH, suggesting that it inhibited the activity of DNMT in a SAH-competitive manner (Castellano et al., 2011).

Laccaic acid A (**63**) is a natural product from the red scales of the insect *Kerria lacca*, which was found to be a new class of anthraquinone-based DNMTi. Laccaic acid A could bind to DNMT1 directly and exhibit 2.5-fold higher potency at DNMT1 inhibition activity than SGI-1027 with a Ki and IC_50_ of 310 and 650 nM (Figure 10), respectively. Notably, it also inhibited the activity of DNMT3. Further research showed that it is competitive with the DNA substrate in methylation assays and alters the expression of methylated genes in MCF-7 breast cancer cells synergistically with 5-aza-2-deoxycytidine. In conclusion, laccaic acid A is a DNMT1 inhibitor with a DNA-competitive manner, which suggests that specifically substituted anthraquinones may be a useful scaffold for DNMTis (Fagan et al., 2013).

Nanaomycin A (**64**) is an antibiotic that was recently reported to selectively inhibit DNMT3B with an IC_50_ of 500 nM, while the activity of DNMT1 was not affected (Figure 10). Nanaomycin A showed anti-proliferation activity in HCT116, A549, and HL60 tumor cell lines with an IC_50_ of 400, 4,100, and 800 nM by reducing the global methylation levels of the RASSF1A gene. Nanaomycin A does not degrade DNMT1 or DNMT3b, indicating that it is a non-covalent inhibitor different from the nucleoside DNMTi (Kuck et al., 2010). However, it is debatable whether the anthracycline group is a good candidate for clinical drug testing due to some cardiotoxicity issues (Horenstein et al., 2000). Nanaomycin A, the first DNMT3B-selective compound, provides a valuable biochemical tool and benchmark for future studies. The antibiotic mithramycin A (MMA) is a potential DNMT1 inhibitor, which could reduce the CpG island methylation of SLIT2 and TIMP-3 genes and could cause the degradation of DNMT1 in lung cancer cells. MMA is recognized as a CG-rich DNA-binding agent, implying that it might also have a similar inhibitory effect against DNMT as procaine (Lin et al., 2007).

Parthenolide (**65**), a sesquiterpene lactone of feverfew, could inhibit the activity of DNMT1 with an IC_50_ of 3.5 μM (Figure 10). The inhibitory activity of parthenolide against DNMT1 may be due to two reasons. Firstly, the electrophilic α-methylene γ-lactone of parthenolide could cause cysteine alkylation of DNMT1. Secondly, it could also down-regulate the expression of DNMT1 by blocking the binding of transcription factor Sp1 to the DNMT1 promoter. A study has shown that parthenolide reversed the methylation of the HIN1 gene and induced re-expression in MCF-7 breast cancer cells. It also induced global DNA hypomethylation in MV4-11 cells (Liu et al., 2009b). Parthenolide could be rapidly absorbed by rats and the plasma concentration decreased by half in about 1 h. The bioavailability was limited and was 7.78% in rats.

Ubiquitin-like plant homeodomain and RING finger domain 1 (UHRF1) is involved in the methylation of DNMTs (Liu et al., 2013). Hinokitiol (**66**), a component of essential oils extracted from *Chymacyparis obtusa*, induces DNA demethylation *via* downregulation of the expression of DNMT1 and UHRF1 as a novel DNMTi (Seo et al., 2017) (Figure 10). A study has shown that hinokitiol altered the methylation of 10 hypermethylated genes in colon cancer cells and reactivated the mRNA expression of O6-methylguanine DNMT, carbohydrate sulfotransferase 10, and B-cell translocation gene 4 (Hu et al., 2015). Interestingly, MGMT is a DNA repair enzyme responsible for demethylating O6-methylguanine in normal cells, which repairs the O6-alkylguanine lesions in DNA by transferring the alkyl groups from the O6-position of guanine to its own active center Cys145 residue and restores normal DNA. It can specifically repair the damage of O6 alkyl guanine in DNA sequences and maintain the accuracy and integrity of the genome (Pegg, 2011). However, for tumor cells, abnormally expressed MGMT specifically removes the lesions at the guanine O6 position, resulting in resistance of tumor cells to guanine O6-alkylating agents like TMZ (Gerson, 2002). In conclusion, the anti-tumor activity of hinokitiol is more like a double-edged sword. On one hand, it can exert anti-tumor activity by inhibiting abnormal DNA methylation; on the other hand, it can cause abnormal MGMT expression and lead to drug resistance toward alkylating agents in tumor cells. This suggests that hinokitiol should not be combined with alkylating agents for antitumor therapy in clinical practice, but should be combined with MGMT inhibitors, which could transfer the alkyl group to the active site Cys145 residue of MGMT and lead the inactivation of MGMT (Sun et al., 2018a). Unfortunately, only two MGMT inhibitors have entered clinical trials. It is urgent to develop new MGMT inhibitors for combination with DNMTis. In a study, researchers predicted the MGMT inhibitory activity of 134 base analogs with QSAR and found some purine analogs, such as O6-benzyl-8-bromo-guanine, O6-thenyl-guanine, and O6-(3-aminomethyl) benzyl-guanine which were potent MGMT inhibitors. In addition, the study also found that there are some substructures, such as 2-bromoprop-1-ene, 2-bromobuta-1,3-diene, thiophene, p-tolylmethanol, ≥2 saturated or aromatic heteroatom-containing ring size 6, E-2-nitroethenamine, ≥3 hetero-aromatic rings, p-xylene, and m-xylene, which are essential for the inhibitory activity of the MGMT inhibitor, which may play an important role in guiding the development of new MGMT inhibitors in the future (Sun et al., 2018b).

Rice-specific component γ-oryzanol (**67**), a mixture of ferulic acid ester and several phytosterols, is a potent DNMTi in the striatum of mice, which significantly inhibited the activity of DNMT1 (IC_50_ = 3.2 μM), DNMT 3A (IC_50_ = 22.3 μM), and DNMT 3B (maximum inhibition 57%) at least partly in an SAH-competitive manner (Figure 10). Furthermore, γ-oryzanol decreased the activity of DNMT1 as an antagonist against ERRγ, which regulates the production of DNMT. Oral administration of γ-oryzanol significantly decreased striatal DNA methylation in the promoter of the D2Rs in HFD-fed mice *in vivo* (Kozuka et al., 2017).

## 4 Conclusion and perspectives

DNA methylation is an important epigenetic process that regulates gene expression. Abnormal DNA methylation leads to transcription inactivation and silent expression of tumor suppressor genes, which results in the occurrence and development of cancer. DNMTis can reverse the abnormal DNA methylation process to achieve the purpose of tumor treatment.

Currently, many DNMTis are under development, primarily including nucleoside and non-nucleoside DNMTis according to the chemical structure. Nucleoside DNMTi is a cytosine nucleoside analog that either directly integrates into DNA sequences in the form of triphosphate to inhibit DNA replication or binds to DNMT covalently to degrade DNMT. Since 2004, the representative first-generation nucleosides DNMTi, 5-azacitidine, clofarabine, and decitabine have been approved by the FDA for the treatment of MDS, CMML, and AML. However, the structure of 5-azacitidine is very unstable and easily hydrolyzed and deaminated. In order to improve the stability of 5-azacitidine and decitabine, a large number of second-generation cytidine analogs have been developed. Zebularine, obtained by removal of 4-amino from the pyrimidine of decitabine, was stable in neutral aqueous solution with a longer half-life. NPEOC-DAC, obtained by introducing the amino-protective group NPEOC, can infiltrate DNA and inhibit DNMTs after the hydrolysis of carboxyl esterase 1 in human hepatoma cells. SGI-110 can resist the deamination of cytidine deaminase with better PK and metabolic stability because of the introduction of a phosphodiester bond. The thiocytidine derivatives T-dCyd and 5-aza-T-dCyd are more stable due to the presence of thiodeoxyribose. 5-Fluoro-2′-deoxycytidine, obtained by the introduction of fluorine in the 5-position of the pyrimidine ring with increasing stability, has entered phase II clinical anti-tumor research. CP-4200, an oleic acid derivative of azacitidine, has better membrane permeability. In general, the chemical stability, metabolic stability, and water solubility of the second-generation nucleoside DNMTis have been greatly improved and some of them can even be taken orally. However, both the first and second generation nucleoside DNMTis are characterized by high toxicity, poor selectivity, and low bioavailability because most of them need to be integrated into DNA sequences to play a role.

In contrast, non-nucleoside DNMTi with various chemical scaffolds has attracted great attention (Medina-Franco et al., 2015). Many non-nucleoside DNMTis have been developed through various strategies. RG108 is the first DNMTi discovered through rational drug design, which can inhibit DNA methylation at low concentrations by blocking the active site of DNMT. Local anesthetic procaine and antiarrhythmic drug procainamide are amino benzoic acid DNMTis found based on drug reposition, which can block DNA methylation by binding the CpG island of DNA. Carbazole DNMTi DC-05 and DC-517 are obtained by virtual screening based on the crystal structure of the DNMT–DNMTi complex, which are the first selective inhibitors of DNMT1. In addition, a large number of DNMTis have been found in natural products. EGCG in green tea, genistein and equol in beans, curcumin in turmeric, resveratrol in peanuts, and caffeic acid in honeysuckle have entered clinical research for cancer treatment. Non-nucleoside DNMTis have a variety of chemical structures and different mechanisms to inhibit DNA methylation. Since they do not need to be integrated into DNA sequences, they have lower toxicity than nucleoside DNMTi. However, the non-nucleoside DNMTi has lower efficacy and poor selectivity than nucleoside DNMTis.

There is an urgent need to develop a new generation of DNMTi with high efficacy, selectivity, and bioavailability. However, unclear issues about DNMT and DNMTi hinder the development of selective DNMT1. First, it remains controversial about which DNMT isoform is the best therapeutic target in cancer. Second, the mechanism of different isomers of DNMT in DNA methylation inhibition and whether a compensation mechanism for DNMT with different isoforms existed are not clear. Third, a DNMTi derived from natural compounds is difficult to be modified and optimized. Forth, there are rare reports on the crystal structure of the DNMT–DNMTi complex, which limits the development of DNMTis through structure-based drug discovery. Fifth, the re-expression of genes following hypomethylation of DNA requires the eviction of nucleosomes from gene promoters. Thus, another complicating factor in the development of DNMTi is the contribution of the nucleosome in the expression of genes (Esteller et al., 2010). Sixth, high structural conservation in the catalytic domains of DNMTs poses a big challenge to design selective DNMTis for a specific DNMT isoforms.

The high toxicity of nucleoside DNMTis is determined by their own properties and mechanism of DNA demethylation. Therefore, it is expected that non-nucleoside-selective DNMTis will be the focus of DNMTi development in the future. First, it is an important way to discover novel DNMTis by exploring the structure–activity relationship of non-nucleoside DNMTis reported so far. Second, we can fuse a nucleoside and a non-nucleoside DNMTi together using a fusion strategy based on structural biology. Similarly, one general approach, called multisubstrate adduct, can be applied in DNMTi design, in which the two substrates SAM and cytosine analog are combined into one single molecule. These adjunctive inhibitors are likely to result in targeting both catalytic and cofactor binding sites, which will increase the binding affinity and selectivity of different DNMT isoforms. Third, chimeric DNMTi contains DNA sequence-specific recognition and effect regions; thus, it can inhibit DNA methylation specifically. Forth, DNMTis with a new structure and mechanism can be found by large-scale computer virtual screening. Fifth, fragment-based drug discovery is another critical way for selective DNMTi discovery. Selective DNMTis can be obtained by growing or linking the new scaffolds that could bind to different isoforms of DNMT. Finally, drug reposition has the benefit to reduce the enormous cost in time and resources devoted to novel drugs.

In conclusion, with the identification of DNMT and inhibitor complex structures and the enrichment of DNMTs’ chemical space, specific and highly effective inhibitors targeting DNMTs will emerge in the coming years. Concerning the high complexity of epigenetic regulation mechanisms, DNMTi may not only be identified as potential therapeutic agents targeting DNA methylation but also could be served as chemical probes that contribute to the better understanding of DNA methylation in normal and cancer cells.

## Glossary

AML: acute myeloid leukemia
APL: acute promyelocytic leukemia
DNMT: DNA methyltransferase
DNMTi: DNA methyltransferase inhibitor
CTL: cytotoxic T lymphocyte
MHC-I: major histocompatibility complex I
KIR: killer cell immunoglobulin-like receptor
MDS: myelodysplastic syndrome
MS: multiple sclerosis
CMML: chronic myelomonocytic leukemia
OR: overall response
CR: complete response
OS: overall survival
PR: partial response
PK: pharmacokinetics
PD: pharmacodynamic
PFS: progression-free survival
PCNA: proliferating cell nuclear antigen
DLT: dose-limited toxicity
EGCG: epgallocatechin gallate
SAH: S-adenosyl-homocysteine
TSG: tumor suppressor gene;
SAM: S-adenosyl-L-methionine
TAAs: tumor-associated antigens
MTD: maximum tolerated dose
Cmax: peak concentration
Tmax: time to maximum plasma concentration
T1/2: half-life
AUC: the area under the curve
Vd: volume of distribution
NPEOC: 2'-deoxy-N4-[2-(4-nitrophenyl)ethoxycarbonyl]
MAGE: melanoma-associated CT antigen
DAPK1: death-associated protein kinase 1
TNBC: triple-negative breast cancer
EMT: epithelial mesenchymal transition
hTERT: human telomerase reverse-transcriptase
TIMP3: tissue inhibitor of matrix metalloproteinase 3
hENT1: human equilibrative nucleoside transporter 1
HDAC: histone deacetyltransferase
PDX: patient-derived xenograft
HKMT: histone lysine methyltransferase
HKDMT: histone lysine demethylase
